# Paediatric sepsis-associated encephalopathy (SAE): a comprehensive review

**DOI:** 10.1186/s10020-023-00621-w

**Published:** 2023-02-23

**Authors:** John Sieh Dumbuya, Siqi Li, Lili Liang, Qiyi Zeng

**Affiliations:** Department of Paediatrics, Zhujiang Hospital of Southern Medical University, Guangzhou, 510282 People’s Republic of China

**Keywords:** Sepsis, Sepsis-associated encephalopathy, Pro-inflammatory cytokines, Cerebral autoregulation, Blood–brain barrier, Oxidative stress, Paediatrics, Central nervous system, Apoptosis

## Abstract

Sepsis-associated encephalopathy (SAE) is one of the most common types of organ dysfunction without overt central nervous system (CNS) infection. It is associated with higher mortality, low quality of life, and long-term neurological sequelae, its mortality in patients diagnosed with sepsis, progressing to SAE, is 9% to 76%. The pathophysiology of SAE is still unknown, but its mechanisms are well elaborated, including oxidative stress, increased cytokines and proinflammatory factors levels, disturbances in the cerebral circulation, changes in blood–brain barrier permeability, injury to the brain’s vascular endothelium, altered levels of neurotransmitters, changes in amino acid levels, dysfunction of cerebral microvascular cells, mitochondria dysfunction, activation of microglia and astrocytes, and neuronal death. The diagnosis of SAE involves excluding direct CNS infection or other types of encephalopathies, which might hinder its early detection and appropriate implementation of management protocols, especially in paediatric patients where only a few cases have been reported in the literature. The most commonly applied diagnostic tools include electroencephalography, neurological imaging, and biomarker detection. SAE treatment mainly focuses on managing underlying conditions and using antibiotics and supportive therapy. In contrast, sedative medication is used judiciously to treat those showing features such as agitation. The most widely used medication is dexmedetomidine which is neuroprotective by inhibiting neuronal apoptosis and reducing a sepsis-associated inflammatory response, resulting in improved short-term mortality and shorter time on a ventilator. Other agents, such as dexamethasone, melatonin, and magnesium, are also being explored in vivo and ex vivo with encouraging results. Managing modifiable factors associated with SAE is crucial in improving generalised neurological outcomes. From those mentioned above, there are still only a few experimentation models of paediatric SAE and its treatment strategies. Extrapolation of adult SAE models is challenging because of the evolving brain and technical complexity of the model being investigated. Here, we reviewed the current understanding of paediatric SAE, its pathophysiological mechanisms, diagnostic methods, therapeutic interventions, and potential emerging neuroprotective agents.

## Introduction

Sepsis-associated encephalopathy (SAE) is an acute brain dysfunction that occurs secondary to infection in the body without overt central nervous system (CNS) infection (Catarina et al. 2021; Yang et al. 2020); symptoms that include impaired consciousness, disorientation, cognitive deficiency, convulsions or deep coma (Chen et al. 2020). SAE is also referred to as sepsis-induced brain dysfunction (SIBD) (Orhun et al. 2019), sepsis-associated brain dysfunction (SABD) (Crippa et al. 2018; Czempik et al. 2020), or sepsis-associated delirium (SAD) (Ebersoldt et al. 2007; Chaudhry and Duggal 2014), depending on the context it is being used. However, they are not entirely synonymous per se. For instance, delirium is one of the clinical features of SAE, while SAE is one of the causes of delirium (Chaudhry and Duggal 2014). The term “mixed encephalopathy” has also been postulated to describe the complexity of its pathophysiological mechanisms involved, reflecting pathological remodelling of the vascular system and blood components in the uncontrolled immune response induced by inflammatory cascades turn to affect the brain endothelium and parenchyma (Shulyatnikova and Verkhratsky 2020; Barbosa-Silva et al. 2021), that subsequently leads to neuroendocrine network dysfunction, diffuse neuroinflammation, impaired BBB integrity, neurotoxicity, and autoregulation (Tauber et al. 2021).

The source and aetiology of infections associated or implicated in SAE include biliary tract or intestinal infections, pulmonary infections and respiratory infections (Czempik et al. 2020; Chaudhry and Duggal 2014). The primary pathogens most often identified are Group B streptococcus, Staphylococcus aureus, Streptococcus pyogenes, Escherichia coli, and unspecified gram-positive rods (Sanz et al. 2018; Jenster et al. 2014); compared to those commonly reported in adults (Zhang et al. 2012; Takemoto et al. 2019), though others argued that there is no correlation between SAE and a particular microorganism (Cotena and Piazza 2012).

### Epidemiology

The prevalence of SAE is challenging to predict or ascertain because there are no specific tests or diagnostic criteria to define this condition (Chaudhry and Duggal 2014), mainly due to its various neurological manifestations and other factors that cause brain dysfunction (Czempik et al. 2020). One of these factors in intensive care unit (ICU) settings is sedation, yielding an estimate in adult patients diagnosed with sepsis, progressing to SAE of anywhere between 9 to 76% (Chaudhry and Duggal 2014; Andonegui et al. 2018; Rivera-Lara 2019). In a recent retrospective cohort study of 140 paediatric patients with sepsis and a clinically indicated MRI within 60 days of sepsis, 30 patients had one or more sepsis-related MRI abnormalities with a prevalence of 21%. Sixteen (53%) patients had sepsis-related white matter signal abnormalities; 53% demonstrated sepsis-related ischaemia, infarction, or thrombosis; and 27% showed sepsis-related posterior reversible encephalopathy. The authors concluded that patients with sepsis-related MRI abnormalities were more likely to die before PICU discharge with increased mortality, new neurological disability at PICU discharge and longer PICU length of stay (Becker et al. 2021). Thus, the incidence and prevalence of SAE in paediatric patients are not demonstrated (Table 1) (Sanz et al. 2018). Another recent review reported an incidence of 20% to 40% in adult patients admitted to the ICU with sepsis-developed encephalopathy, with delirium accounting for approximately 70% in mechanically ventilated elderly patients. In comparison, hospitalised septic patients are associated with a 10% increase in the prevalence of cognitive impairment during eight years (Mazeraud et al. 2020). Indeed, such data are urgently needed to help with understanding its epidemiological distribution because paediatric patients seem to suffer more from SAE-associated sequelae, such as attention, verbal fluency, executive function, IQ, school performance, memory acquisition and processing, and quality of life in later years.

**Table 1:** Epidemiology of paediatric SAE

Auther, year	Country	Design	Settings	Sample size	Age	Incidence/prevalence	Mortality	Outcome
Sanz D	UK	Retrospective descriptive study	Tertiary PICU	193	≤ 10 yrs	Incidence 10.3%	28%	Length of mechanical ventilation and length of stay in ICU days were increased; ischaemia and cerebritis were most common brain lesions on MRI
2018								
Jenster M	USA	Prospective cohort study	Intensive care Nursery of the University of California	258 (29)^a^	Neonates	Prevalence 11%	58%	Abnormal cognitive outcome; Watershed injury; Maternal chorioamnionitis have lesser moderate-severe brain injury
2014	San Francisco							
Algebaly A	Egypt	Prospective study	Cairo University Hospital	75	≤ 30 months	N/A	53.30%	High PRISM III score in SAE patients; High PI and RI values in SAE patients; High cerebrovascular resistance; High frequency of vasopressor use and mechanical ventilation
2020								
Becker AE	USA	Retrospective cohort study	Tertiary PICU	140	≤ 18 yrs	Prevalence 21%	7%	White matter signal abnormalities on MRI; New neurological disability at PICU discharge; longer PICU length of stay
2021	Pennsylvania							
Sandquist MR	USA	Retrospective chart review	CCHMC	800 (389)^b^	≤ 12 yrs	N/A	N/A	Volume loss; Numberous, large, bilateral MRI signal and/or CT attenuation abnormalities in the cerebral cortex and white matter; High PRISM III and presence of oncologic diagnosis/organ transplantation were independently associated with abnormal neuroimaging study
2017	Ohio							
Kaur J	India	Prospective study	Tertiary PICU	50	≤ 18 yrs	N/A	N/A	Delayed neurodevelopment; low verbal IQ; Decline in school performance; Low intelligence at short-term follow-up; Poor behavioural outcomes
2016								
Punchak M	Mozambique	Retrospective, observational	Tertiary adademic PICU	987 (182)^c^	≤ 15 yrs	Prevalence 18%	58%	Increased noninfectious CNS pathologies, neoplastic diseases
2018								

*SAE* Sepsis-associated encephalopathy, *CNS* Central nervous system, *CT* Computerised topography, *IQ* Intelligence quotient, *ICU* Intensive care unit, *CCHMC* Cincinnati children's hospital medical center, *PICU* Paediatric intensive care unit, *PRISM III* Paediatric risk of mortality assessment III, *N/A* Not applicable, *PI* Pulsatility index, *RI* Resistivity index, *MRI* Magnetic resonance imaging

^a^Of the 258 patients included in the study, 29 patients were diagnosed with sepsis

^b^Of the 800 patients diagnosed with sepsis/septic shock,, 389 patients had abnormal neuroimaging

^c^Of the 987 patients included in the study, 182 patients were diagnosed with sepsis

The mortality and morbidity of SAE reported in the literature vary according to different studies. For instance, in a prospective case–control study, the authors compared the neurodevelopmental and behavioural outcomes in 50 children with sepsis-associated encephalopathy. They observed that children with SAE had low intelligence at 52% compared to 32% for controls and showed declined school performance at 44%, disobedience at 28%, and stubbornness/irritable behaviour at 26%. They concluded that children with SAE had delayed neurodevelopment, low verbal IQ, a decline in school performance and low intelligence at short-term follow-up (Kaur et al. 2016). Another retrospective study was in Mozambique, Sub-Saharan Africa, where the authors recruited 987 paediatric patients diagnosed with different diseases. Of these, 182 (18%) were diagnosed with sepsis, with a mortality of 56%, which was the highest among all other diseases; the authors found burns at 45%, positive HIV tests at 24%, malaria at 24%, respiratory tract infections at 21% and trauma 6% (Punchak et al. 2018). This might be the tip of the iceberg regarding sepsis mortality in paediatric patients, especially in resource-limited healthcare settings, and probably because of under-reporting.

From those mentioned above, it is conceivable that SAEs have both short-term and long-term mortalities and morbidities with different associated risk factors. Children's most reported short-term mortality was cognitive impairment and poor academic performance (Sandquist et al. 2017). The long-term sequelae reported in the general population include physical, cognitive, and psychological impairment with high socioeconomic burdens (Ehler et al. 2017); further sequelae were anxiety, stress disorders, and lower quality of life (Orhun et al. 2019); memory lapse, inattentiveness, disorientation, and verbal difficulties (Nwafor et al. 2019). Other comorbidities include hypertension, anaemias, and neurological diseases other than SAE (Yang et al. 2020; Chen et al. 2020). Underlying conditions also pose a high risk of SAE occurrences, such as renal failure and metabolic disturbances (hypo/hyperglycaemia, hypercapnia, hypernatremia) (Sonneville et al. 2017). Children are more susceptible to metabolic derangement often encountered in the PICU, thus making them more prone to SAE development than adults. However, very few documented paediatric SAE incidences and mortalities are probably due to the few cases reported in the literature or the underreported incidence rate.

## Diagnosis

The diagnosis of SAE involves excluding direct CNS infection or other types of encephalopathy (Czempik et al. 2020; Huang et al. 2020), which hinders its early detection and appropriate implementation of management protocols, thus resulting in its associated high mortality rate. This scenario becomes more evident in paediatric patients than adults, with few documented cases (Table 2). Clinical assessment, electrophysiological, neurological imaging and biomarkers are employed to aid diagnosis and to direct therapeutic strategies. However, most of these diagnostic tools are potentially hampered by sedation and mechanical ventilation, thus delaying appropriate intervention strategies. Below we described the most commonly applied diagnostic paradigm in suspected septic patients.

**Table 2:** Characteristics of reported cases in paediatric SAE

Case (author)	1 (Chacqueneau AL 2013)	2 (Abe S 2018)	3 (Takemoto R 2019)	4 (Huang L 2020)	5 (Kondo A 2009)	6 (Kondo A 2009)
Age (sex)	4 years, (F)	2 months, (F)	2 years, (F)	5 years, (F)	2 years 9 months, (F)	17 months, (M)
Clinical features	Fever, vomiting, vigilance disorders, loss of verbal fluency, cerebral syndrome	White stool, icterus, hepatosplenomegaly; seizures, mild left hemiplegia (1st week)	Ankle injury due to accident; 6 m later: fever, general fatigue, localised left ankle pain, GTCS	Abdominal pain, vomiting, coma, tachypnoea, tachycardia	Coma, seirues	coma, seizures
Initial diagnosis	Infectious encephalitis	Biliary atresia	Bicycle spoke injury	NORSE	N/A	N/A
EEG findings	Globally slow waves	Mild activity in the right hemisphere	Poorly organised background activity, slow waves	Generalised diffuse and slow background activity	Poor background activity, global slow waves	Poor background activity
MRI findings	Diffuse brain oedema with extented involvement of the cortical and basal ganglia	Abnormal intensity in the subecortical white matter of the frontal lobe and occipital region	T2-prolonged lesions in the mesial frontal cortex, reduced diffusion on DWI	High signal intensity in the periventricular white matter on DWI	Restricted diffusion in the basal ganglia, subcortical white matter in the frintal and occipital lobes	Cracked lesions in the white, matter, brainstem and cerebellum
Other test	Increased CRP and procalcitonin levels	Elevated LFT and bilirubin levels; CT: low density in the right hemisphere	Increased CRP and procalcitonin, nucleated cells on CSF	Elevated CRP and procalcitonin, abnormal coagulants, abnormal LFT; tachycardia on ECG; bronchitis on X-ray	CT: Diffused brain edema Angiography: Diminished intracranial major arteries	CT: Diffused brain edema Angiography: Diminished intracranial major arteries
Final diagnosis	SAE	SAE	SAE	Hyperferritinemic sepsis	SAE	SAE
Treatment	Antibiotic and surgery for perforated appendicitis	Midazolam;glycerol, iv dexamethasone; liver transplant	Antibiotics, IVIG, edaravone	Antibiotics, TPE	Fluids, antibiotics	Fluids, antibiotics
Follow up	All exams normal at 1 year	MRI: mild atrophic changes 4 weeks after discharge; normal neurological function at 19 months	Normal MRI on day 10, normal neurological function 2 weeks after discharge	T2/FLAIR: high diffuse intensity in the white matter 17 d after onset; transferred to rehab center 21 d after onset; full recovery at 55 d	Brain death on day 73	Severe disability one and a half years later

*CRP* C-reactive protein, *CT* Computer topography, *GTCS* Generalised tonic–clonic seizures, *MRI* Magnetic resonance imaging, *LFT* Liver function test, *DWI* Diffusion weighted imaging, *EEG* Electroencephalography, *ECG* Electrocardiography, *FLAIR* Fluid attenuated inversion recovery, *IVIG* Intravenous immunoglobin, *NORSE *New-onset refractory status epilepticus, *N/A* Not applicable, *SAE* Sepsis-associated encephalopathy, *TPE* Therapeutic plasma exchange, *CSF* Cerebrospinal fluid

## Electrophysiological tools

### EEG

The most commonly used diagnostic tool is electroencephalography (EEG), which measures spontaneous electrical activity generated by synaptic transmission in the superficial layers of the cerebral cortex and modulated by subcortical structures from the upper brainstem to the thalamus (Hosokawa et al. 2014). The severity of EEG is classified into excessive θ, predominant δ, or triphasic waves, and suppression or burst suppressions (Chen et al. 2020; Tsuruta and Oda 2016). The changes observed in these waveforms correspond to the changes in brain function. In other words, slow alpha activity and increased theta activity are associated with cortical dysfunction, often observed in encephalopathic patients; slow delta activity indicates an impaired function of the deeper brain structures associated with more severe neurocognitive decline (Nwafor et al. 2019), and burst-suppressions are associated with severe symptoms (Czempik et al. 2020) and poor prognosis (Hosokawa et al. 2014). The mortality is also related to the severity of EEG abnormalities, ranging from 19 to 67% (Chaudhry and Duggal 2014). In addition to its feasibility and accessibility in most ICU tertiary institutions, EEG has high sensitivity in diagnosing SAE patients and its associated complications; it is also valuable for excluding non-convulsive status epilepticus in critically ill patients caused by altered sensorium (Pantzaris et al. 2021). However, its specificity is very low and hampered by sedatives, making its interpretation inconclusive in severe cases (Ebersoldt et al. 2007; Ehler et al. 2017). EEG manoeuvre is also very challenging, especially in ventilated children. Reduced hippocampal volume and memory deficits in SAE patients might not show abnormality in the EEG during hospitalisation, implying that sepsis leads to damage to specific regions of the hippocampus undetectable by EEG (Yuan et al. 2020).

### SEP

Another electrophysiological tool is sensory evoked potentials (SEPs), which have recently gained popularity in diagnosing SAE. Evoked potentials (EPs) measure brain responses to sensory stimulation, including responses generated by subcortical structures (brainstem auditory evoked potentials (BAEPs) or from N14 and P18 somatosensory evoked potentials (SSEPs)) (Hosokawa et al. 2014). SEP show peak latencies in cortical and subcortical pathways and is associated with SAE severity (Cotena and Piazza 2012; Tsuruta and Oda 2016). It is not affected by sedation, as an advantage, while its drawbacks are that it is cumbersome to use in ICU settings and possibly expertise availability and interpretation.

Thus, electrophysiological tools may aid in the early clinical assessment of suspected SAE patients and help guide treatment strategies, but considering its associated limitations (especially sedation, which is equivocally unavoidable in ICUs for agitated patients) make its diagnostic accuracy unreliable that warrant further validation. Thus, other diagnostic modalities are needed to increase diagnostic accuracy and early treatment strategy initiation.

## Biomarkers

Serum biomarkers are routinely employed in patients admitted to ICU to help assess the severity of brain injury, not only in cases with encephalopathies but also in traumatic brain injury and stroke (Wu et al. 2020). Such biomarkers include NSE, S100B, and GFAP.

### NSE

Neuron-specific enolase (NSE) is a gamma-enolase isomer of the cytoplasmic glycolytic enzyme found in neurons and neuroendocrine cells (Zenaide and Gusmao-Flores 2013). A high concentration of NSE and S100B are associated with SAE severity. S100B correlates strongly with severe encephalopathy and other brain lesions than the other biomarkers (Cotena and Piazza 2012). Zhang et al. assessed the expression levels of S100B, NSE, and GFAP in paediatric septic patients; their results showed higher levels of serum NSE, S100 β and GFAP than that of controls and that NSE and S100 β were the highest in children who did not survive sepsis (Zhang et al. 2014). In another study in children with septic encephalopathy, the authors evaluated serum intercellular adhesion molecule-1 (ICAM-1), nitric oxide (NO), lipid peroxide (LPO) and S100B. They demonstrated elevated levels of these biomarkers, not only in serum but also in cerebrospinal fluid (Hamed et al. 2009).

In contrast, Zhu et al. 2016 reported that NSE and IL-6 demonstrated the more diagnostic significance of SAE than S100B (Zhu et al. 2016). This discrepancy might be due to diagnostic methods and cut-off values used in each study. NSE in cord blood and cerebral blood flow (CBF) in early-onset neonatal sepsis (EONS) were examined to predict SAE occurrence and showed that increased cord blood NSE and CBF in early hours of birth could be used in neonates with EONS with a predictive accuracy of SAE (Shimy et al. 2018).

### GFAP

Glial fibrillary acidic protein (GFAP) is also increased in SAE patients with specificity and sensitivity of 77.7% and 75.9%, respectively; here, serum GFAP level correlated positively with APACHE II score but negatively correlated with Glasgow Coma Scale (GCS) score, 28- day survival rate and 180-day survival rate (Yan et al. 2019). The serum concentration of GFAP and ubiquitin C-Terminal hydrolase-L1 (UCH-L1) were assessed in SAE patients where GFAP was associated with worse long-term usual activities, and UCH-L1 had more long-term pain (Wu et al. 2020). However, the clinical significance of elevated S100B and NSE levels in SAE patients has been questioned due to their poor sensitivity and specificity (Spapen et al. 2010). Nevertheless, these studies have demonstrated the significance of these biomarkers in diagnosing sepsis and other infectious diseases, pointing to the need for more research to validate their potential diagnostic accuracy in sepsis or SAE.

Other biomarkers include Neurofilament (Nf) (Ehler et al. 2019; Manabe and Heneka 2021), S100A8 protein (Hamasaki et al. 2019), Amyloid β peptide and tau proteins (Zhao et al. 2019), vascular cell adhesion molecule-1 (VCAM-1) (Su et al. 2014), and acetylcholinesterase activity (Zujalovic et al. 2020). Others have suggested using some microRNAs as a marker for diagnosing SAE as they play a central role in the pathophysiological processes of SAE (Osca-Verdegal et al. 2021). For instance, miR-370-3p was increased in the brain and plasma of SAE mice induced by LPS (Visitchanakun et al. 2020). MiR-29a is highly expressed in the peripheral blood of patients diagnosed with SAE and can be used as a molecular marker for early diagnosis and prognostic prediction of SAE patients (Guo et al. 2021). Noninvasive bedside monitoring through physical examination is also essential in aiding the early detection and management of patients with sepsis (Postelnicu and Evans 2017). These noninvasive parameters include mental status changes, capillary refill time (CRT), skin mottling and temperature gradients. These manoeuvres are very useful in paediatric ICUs though they heavily depend on the clinician’s expertise.

These biomarkers need further study to validate their diagnostic accuracy, especially in paediatric patients with sepsis-related encephalopathies, to aid early diagnosis and implementation of appropriate interventions to decrease mortality and improve neurological outcomes associated with SAE.

## Neurological imaging

### MRI

Magnetic resonance imaging (MRI) is the ICU's most commonly used neurological imaging modality. Different parameters of MRI have been used, including diffusion-weighted imaging (DWI), apparent diffusion coefficient (ADC), and fluid-attenuated inversion recovery (FLAIR). Their common abnormalities include multiple ischaemic strokes or white matter lesions in the centrum semiovale (Kuperberg and Wadgaonkar 2017). Sandquist et al. 2017 identified 80 abnormal MRI findings in their cohort study. They observed that the most common were abnormal hyperintense signals on T2 in 46 patients and 40 patients on FLAIR; the most common sites were white matter and cerebral cortex (Sandquist et al. 2017). In an animal model, Bozza et al. observed that a decreased ADC is more evident in animals that would not survive the septic challenge than in surviving animals (Bozza et al. 2010).

In one study involving 194 children diagnosed with septic encephalopathy, the predominant watershed pattern of injury was the most common pattern, seen in 98 (38%) newborns, whereas 59 (23%) showed the basal ganglia/thalamus as the predominant pattern on brain MRI (Jenster et al. 2014). Another observational study found that ischaemia and cerebritis were the most frequent brain lesion patterns on neuroimaging, with volume loss as the most common abnormal findings in paediatric patients (Sanz et al. 2018). In a case report by Chacqueneau et al. 2013, their patient's MRI showed non-specific diffuse lesions with vasogenic oedema on the subcortical substance or the basal ganglia and the thalami (Chacqueneau et al. 2013). While Abe et al. 2008 reported an abnormal intensity in the subcortical white matter of the frontal lobe and occipital regions in 2 month old diagnosed with SAE (Abe et al. 2008). Kondo et al. 2009 also reported similar findings in two patients diagnosed with SAE (Kondo et al. 2009). BBB dysfunction, impaired vascularity, and decreased brain metabolites were demonstrated as a measure of long-term neuroinflammatory indicators when assessed by MRI and MRS in the LPS-induced rat SAE model (Towner et al. 2018). However, the downside of this modality is its cost and the risk of transporting critically ill patients, limiting its use in the clinical management of septic or SAE patients (Cotena and Piazza 2012).

### Transcranial Doppler

Another commonly used modality is Transcranial Doppler (TCD) which assesses vasomotor activity (Lamar et al. 2011). As with MRI, TCD also has different parameters used to assess cerebral blood flow (CBF) and fluid volume (FV) in cerebral arterioles, such as pulsatility index (PI) and resistive index (RI). A study by Algebaly et al. recruited 45 children with SAE and found that PI and RI were significantly higher in SAE patients compared to their counterparts without SAE. Specifically, PI was more negatively correlated to a Full outline of unresponsiveness (FOUR) score with high significance and PI related well with illness severity when assessed by the paediatric risk of mortality assessment III (PRISM III) and hence increasing cerebrovascular resistance (CVR) with subsequent deepening of coma (Algebaly et al. 2020). Similar results were also reported in SAE adult patients, where a majority of patients (76%) presented a maximum PI > 1.1, showing a lower GCS at the initiation of sepsis and indicating that a PI cut-off value of > 1.3 could be used in clinical practice as a risk factor for delirium in septic patients (Pierrakos et al. 2014). However, one study reported variability of TCD measurement attributable to age, sedation, and arterial partial pressure of carbon dioxide (PaCO2) (Pfister et al. 2008), making definitive estimation of average range flow volume (FV) a challenge.

### ScvO2 and rSCO2

One study recorded changes in central venous oxygen saturation (Scvo2) and regional cerebral oxygen saturation (rSco2) in children with SAE at different time points to determine prognosis and its related clinical features. The results showed that the ScvO2 values in the deceased group were significantly higher than those in the survivors' group at all different time points. The differences were statistically significant, suggesting that changes in ScvO2 are closely related to the prognosis of children with sepsis or SAE (Guo et al. 2019). The authors suggested that the importance of continuous monitoring of Scvo2 changes with other measurement modalities in evaluating treatment is closely related to the prognosis of children with SAE.

Other diagnostic methods in adult animal models include intravenous acetazolamide to assess cerebral vasomotor reactivity (VMR) using maximal cerebrovascular reserve capacity (CRC) in patients with SAE (Szatmári et al. 2010). Nuclear medicine radiotracers have also been used in SAE models (Szöllősi et al. 2018). At the same time, another study used Gas Chromatography-Mass Spectrometry (GC–MS) to find the differences in plasma metabolites in SAE patients that were strongly correlated in predicting SAE severity when assessed by GCS (Zhu et al. 2019). Measurement of optic nerve sheath diameter was used to detect intracranial hypertension (ICH), a common risk factor associated with SAE (Yang and Sun 2020; Czempik et al. 2020; Wang et al. 2022), and histopathological changes (Shulyatnikova and Verkhratsky 2020).

Brain CT is also routinely applied, showing diffuse oedema of the whole brain (Sanz et al. 2018; Guo et al. 2019). However, it is less applicable due to its associated radiation effect. Another study postulated that near-infrared spectroscopy (NIRS) could be used to identify blood pressure ranges that enhance autoregulation in patients with SAE and that disturbances in autoregulation are associated with the severity of encephalopathy (Rosenblatt et al. 2020). Large clinical trials are needed to validate these emerging diagnostic tools and the efficacy of the investigated drugs to help clinicians and healthcare providers with a more robust approach to treating and managing SAE patients.

Nomograms are being developed for early identification and stratification of appropriate treatment and predicting hospital mortality, risk factors and prognosis in SAE patients (Yang et al. 2020; Zhao et al. 2021).

## Pathophysiological mechanisms

**Fig. 1:**
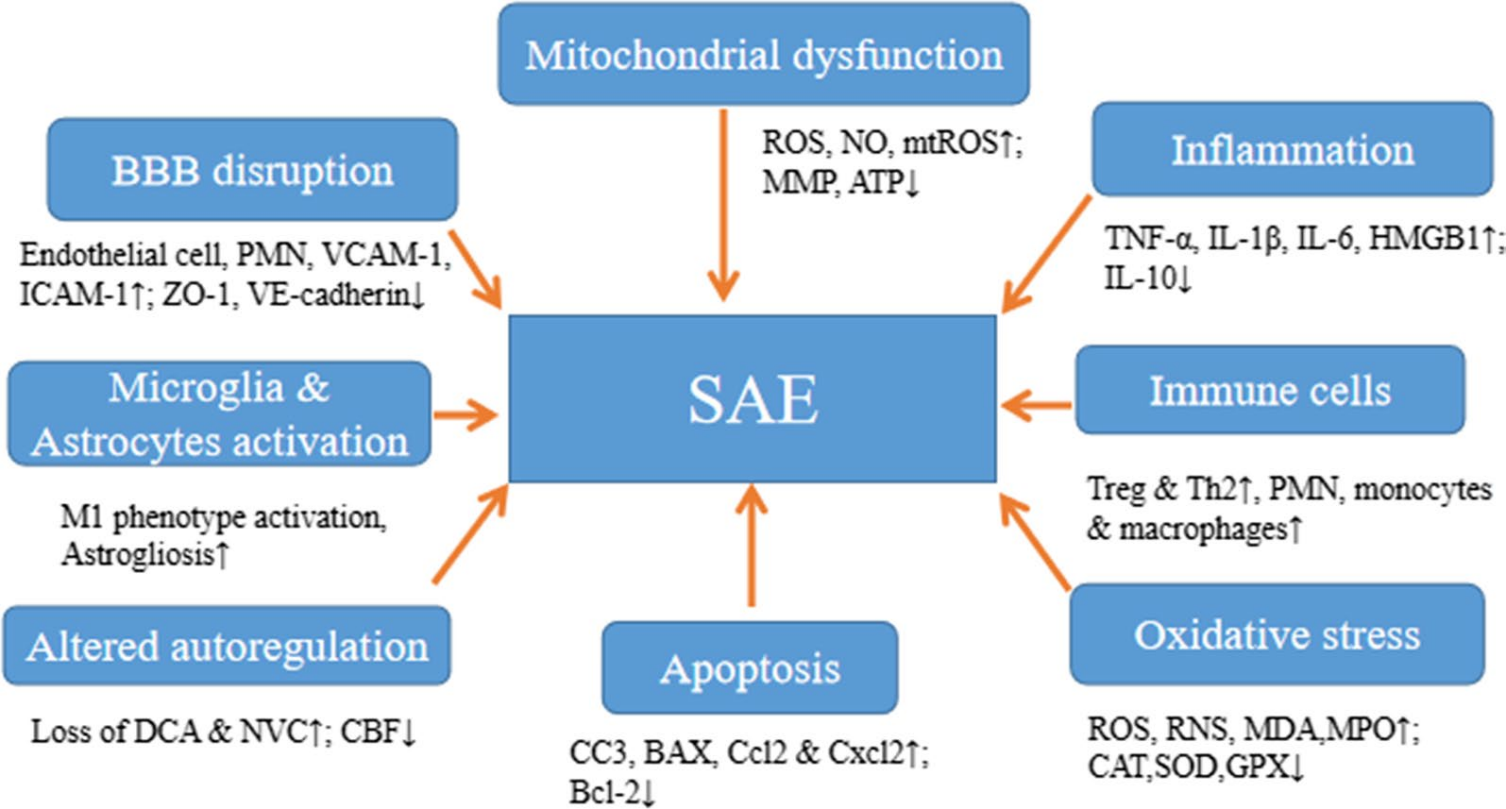
Proposed pathophysiological mechanisms involved in SAE. *SAE *Sepsis-associated encephalopathy, *TNF-α* tumour necrosis factor alpha, *IL-1β* Interleukin 1beta, *HMGB1* High-mobility group box 1, *VCAM-1* Vascular cell adhesion molecule 1, *ICAM-1*: Intercellular adhesion molecule 1, *SOD* Superoxide dismutase, *CAT* Catalase, *NO* Nitric oxide, *ROS* Reactive oxygen species, *RNS* Reactive nitrogen species, *PMN* Polymorphonuclear cells, *mtROS* mitochondrial reactive oxygen species, *GPX* Glutathione peroxidase, *MDA* Malondialdehyde, *MPO* Myeloperoxidase, *MMP *Mitochondrial membrane potential, *DCA* Dynamic cerebral autoregulation, *NVC* Neurovascular coupling, *CBF* Cerebral blood flow, *ZO-1* Zonular occludens 1, *CC3* Cleaved caspase 3, *Tregs* Regulatory T cells, *Th2* helper T cells

The mechanisms of SAE are well established (Fig. 1), ranging from oxidative stress, increased cytokines and proinflammatory factors levels, disturbances in the cerebral circulation, changes in blood–brain barrier permeability, injury to the brain’s vascular endothelium, altered levels of neurotransmitters, changes in amino acid levels, dysfunction of cerebral microvascular cells, mitochondrial dysfunction, activation of microglia and astrocytes, and neuronal death (Chen et al. 2020; Ziaja 2013), while its pathophysiology remains unclear (Crippa et al. 2018). The most described mechanisms include microcirculatory dysfunction; BBB impairment; cerebral autoregulation disruption; inflammatory cytokine activation, and oxidative stress. These pathogenetic mechanisms have similar characterisations in adults and children, though their pathogenesis and clinical presentation might differ due to the evolving brain. For instance, systemic adaptive and innate immune responses following infections react differently between adults and neonates, as helper type Π (Th2) cells tend to favour neonates in their function. In contrast, Th1 cells function more in adults (Brochu et al. 2011). The immature brain is also more resistant to injury, possibly due to a lower cerebral metabolic rate, the plasticity of immature CNS, and immaturity in the development of balance in the available neurotransmitters (Vaishali and Patel 2014). Functional BBB response also differs after brain injury insult, as well as differences in gene expression of cerebral endothelial cells (Zhang et al. 2019). In addition, sepsis-related brain damage in children is more or less of vascular regulation dysfunction rather than direct damage from infectious agents (Sanz et al. 2018), probably due to the immaturity of the developing brain. Therefore, these differences might influence how these pathogenetic mediators respond to specific brain injury insults in adults and children. Below, we describe the most implicated pathogenetic mechanisms activated or disrupted following sepsis or SAE. It should be noted that these mechanisms may act independently or in synergy to induce pathophysiological changes seen in patients diagnosed with SAE.

## Dysregulation of inflammatory cytokines

Pro-inflammatory cytokines (PICs) are activated following an infection, such as tumour necrosis factor-alpha (TNF-α), interleukin-1 beta (IL-1β), and IL-6. Infiltration of these cytokines, in turn, enhances the activation of endothelial cells and microglia, ultimately leading to the loss of neuronal function (Nwafor et al. 2019). These cytokines also modulate the expression of AMPARs and N-methyl-D-aspartate receptors (NMDARs) on neurons, further causing aberrant neuronal function and resulting in delirium and SAE. Different routes and regions of the brain are accessible by inflammatory signals through neural or humoral pathways that will trigger inflammatory stress responses clinically observable in sickness symptoms (Moraes et al. 2021).

Upregulation of PICs genes is also involved in microcirculatory dysfunction by potentially altering blood flow (Szatmári et al. 2010). Upregulation of TNF-α mediates SAE occurrence due to its direct correlation with BBB disruption, brain oedema, neutrophil infiltration, astrocytosis, and apoptosis of brain cells, but not in TNFR1-deficient mice (Ren et al. 2020). The mRNA expression of TNF-α and its receptor, TNFR1, is upregulated following LPS induction in the septic encephalopathy model (Alexander et al. 2008). IL-1β activates afferent vagal fibres in the nucleus tractus solitarius, further causing cerebral damage and stimulating the hypothalamic–pituitary–adrenal (HPA) axis (Ebersoldt et al. 2007). TNF-α and IL-1β activation can induce IL-6, cyclooxygenase 2 (COX2), implicated in activating the HPA axis (Cotena and Piazza 2012). Stimulated microglia and astrocytes by cytokines produce other cytokines, chemokines, nitric oxide, excitatory amino acids, COX2, and reactive oxygen species (ROS), which are detrimental to the immature brain due to the enhanced vulnerability of maturing cells (Bartha et al. 2004). Inflammatory cytokines also disrupt the blood-cerebrospinal fluid barrier (BCSFB) by increasing the influx of peripheral neutrophils through the choroid plexus in a neonatal infection leading to SAE (Michels et al. 2015). Activated cytokines can inhibit the differentiation and proliferation of oligodendrocyte precursors, which affects active myelination and may lead to white matter injury (Bartha et al. 2004), commonly seen in paediatric brain injury.

One study compared expression levels of PICs in both preterm and term septic rat models; the authors reported that at postnatal day 1 (P1), the neuroinflammatory reaction triggered by hypoxia–ischaemia (HI), lipopolysaccharide (LPS) or LPS + HI was limited to IL-1β and monocyte chemoattractant protein 1 (MCP-1) with no TNF-α over-expression, which was more prominent within the cerebral white matter than in the grey matter. At the same time, anti-inflammatory cytokines' responses were absent (IL-6, IL-10) or even down-regulated (IL-1ra, TGF-β1) under HI, LPS or LPS + HI conditions. In contrast, at p2, both pro-and anti-inflammatory cytokines were over-expressed within brains exposed to HI or LPS + HI. The authors concluded that p1 is more vulnerable to neurotoxicity than p2 due to the immaturity of neuronal cells (Brochu et al. 2011). From this study, it is conceivable that children respond differently to brain injury insults and are more susceptible than adults. 'Cytokine storm' has also been implicated in the pathogenesis of SAE, where PICs decrease nitric oxide (NO), resulting in cerebral arteriolar resistance and decreasing CBF and blood volume (Czempik et al. 2020). However, other studies have reported opposing results regarding cytokine activation during inflammatory response (Orhun et al. 2019; Andonegui et al. 2018; Blom et al. 2015). Nevertheless, these studies have shed more light on the pathophysiological understanding of SAE due to the disruption of these inflammatory mediators.

## BBB disruption

Another critical element in the pathogenesis of SAE is blood–brain barrier (BBB) impairment, which regulates the microenvironment of the nervous system, controls blood flow through the brain capillaries, and protects against the influx of harmful substances circulating in the blood (Ziaja 2013). The BBB comprises endothelial cells, astrocytes, pericytes, and basal lamina (Nwafor et al. 2019). Brain microvascular endothelial cells (BMVELs), the main constituent of BBB, are located in close apposition to perivascular pericytes, astrocyte foot process, and microglia. Its structural support is provided by cellular adhesion molecules (CAMs) and transmembrane proteins, including junctional adhesion molecules, claudins, and the adaptor cytoplasmic proteins zonula occludens-1–3, which connect to the actin cytoskeleton and serve as a scaffold as well as mediate cell–cell interactions (Kuperberg and Wadgaonkar 2017). Increased BBB permeability is caused either by endothelial activation or astrocyte end-foot swelling that results in vasogenic oedema (Cotena and Piazza 2012). The disruption of BBB integrity can lead to numerous cytokines and leukocyte infiltration in brain tissue, causing neuronal apoptosis and dysfunction (Kuperberg and Wadgaonkar 2017; Peng et al. 2021). Endothelial cells and polymorphonuclear (PMN) activation result in BBB breakdown, which may lead to dysfunction of the brain's extracellular environment and subsequent neuronal dysfunction, resulting in SAE (Blom et al. 2015). Endothelial cell injury caused by neuroinflammation further leads to derangement of cerebral perfusion, which renders the ischaemic processes of SAE an intractable problem (Ren et al. 2020). Activated endothelial cells not only lead to BBB breakdown but also alter microcirculation and vascular tone, changes that can lead to ischaemia or haemorrhagic lesions (Lamar et al. 2011).

The pathophysiological factors implicated in BBB disruption during the pathogenesis of sepsis include upregulation of vascular endothelial growth factor (VEGF) and activation of VEGFR2, disorganisation of adherens junctions, reduced expression of tight junction proteins, activation or upregulation of inflammatory cytokines, oxidative stress induction and upregulation of matrix metalloproteinases (Archie et al. 2021). These factors may act in synergy to disrupt BBB permeability, further exacerbating SAE pathology, resulting in neuroinflammation, neuronal degradation and potentially cell death with resulting sepsis-induced brain dysfunction. Recent studies have shown that the choroid plexus and circumventricular organs (CVOs) are more permeable to inflammatory mediators that cross the BBB or signals via neurovascular units (NVUs) (Moraes et al. 2021). Therefore, one of the ways inflammatory cytokines enter the BBB other than the active transport via specific carriers is through the vulnerable CVOs that lack BBB but contain neuronal cells that receive chemical input from the bloodstream, both endogenous mediators of inflammation and pro-inflammatory products of pathogens (Tauber et al. 2021).

There is a decrease in branch chain amino acids (BCAAs) and an increase in aromatic amino acids (AAA) in septic encephalopathic patients, and their ratio is significantly increased due to the disruption of BBB integrity (Chaudhry and Duggal 2014). Reduced autothermia temperature (Ta) was associated with early BBB breakdown in LPS-induced SAE, where mice kept at reduced Ta developed an exacerbated and prolonged hypothermia when Ta was reduced from 3 ℃ to 4 ℃ below the lower critical Ta value (Lang et al. 2020). However, in a rat model of SAE, Griton et al. showed that SAE could occur without BBB breakdown. Instead, it increases water diffusion anisotropy, alters glial cell morphology in brain white matter, and decreases expression of COX-2 and AQP4 in the cortex, suggesting that early SAE is related to changes in cerebral blood flow and white matter microstructure instead of BBB breakdown (Griton et al. 2020). Another study reported that the BBB is relatively resistant to LPS-induced disruption, with some brain regions more vulnerable to LPS (hippocampus, thalamus, pons-medulla) than others (Hypothalamus, occipital cortex), with increased BBB permeability at a dose of 3 mg/kg of LPS and no effect at a dose of 0.03 mg/kg or 0.3 mg/kg; and that this disruptive effect is probably dependent on COX rather than oxidative stress (Banks et al. 2015). Thus, though an essential mechanism in the pathogenesis of SAE, BBB disruption might not be the sole culprit in SAE pathophysiological changes.

## Microglia and astrocytes activation

The functions of microglia include surveillance, neuroprotection, phagocytosis, and toxicity. Recent studies showed that astrocytes and microglial cells are involved across the SAE pathomechanistic spectrum during SAE inflammatory activation, with inflammatory activation occurring mainly in microglial cells (Moraes et al. 2021). Thus, microglial activation is one of the key players in neuroinflammation implicated in SAE pathophysiological processes.

Microglia activation involves two phenotypes, M1 cells that produce PICs and ROS, causing neurotoxicity and M2 cells that produce anti-inflammatory effects that play a neuroprotective role and a tissue repair function (Moraes et al. 2021; Michels et al. 2015). Thus, microglial activation acts as a double-edged sword where M1 activation-induced release of inflammatory mediators causes neurotoxicity, while M2 activation results in neuroprotection. Activated microglia deteriorate BBB integrity, subsequently enhancing ROS release, which leads to brain dysfunction (Ren et al. 2020). Activated microglia can affect the amygdala leading to obvious psychological manifestations in septic patients (Czempik et al. 2020). Microglia depletion during severe sepsis development is associated with early exacerbation of brain and systemic inflammation (Michels et al. 2019). Microglial activation is an early responder during the acute phase of neuroinflammation. However, recent studies have demonstrated that cerebral endothelial cells (CECs) are the most likely initial source of inflammatory mediators with subsequent activation of apoptotic signalling that will lead to BBB disruption resulting in leakage of peripheral cytokines into the CNS, exacerbating the vicious neuroinflammatory cascade, implicated in the pathophysiology of SAE (Kodali et al. 2021). Prolonged soluble epoxide hydrolase (sEH) reactivity in CECs may be one of the culprits (Wang et al. 2020).

Similarly, astrocytes, which control homeostasis and catabolism, also have two forms; reactive astrogliosis triggers nervous tissue damage by attracting immune cells specifically to the injured region and facilitating their extravasation and tissue infiltration (Shulyatnikova and Verkhratsky 2020). Astrocytes are not only involved in CBF by controlling the release of mediators activated following inflammation, but they also regulate the concentration of neurotransmitters, such as glutamate, GABA, and glycine, in the synaptic space by taking up any excess neurotransmitter, that will further exacerbate neuroinflammation resulting in long-term consequences of SAE (Barbosa-Silva et al. 2021; Mazeraud et al. 2020; Heming et al. 2017). Thus, astrocytes' activation may lead to an uncontrollable release of these mediators and the overwhelmed concentration of neurotransmitters and subsequent accumulation of these neurotransmitters into the synaptic space, disrupting synaptic space integrity.

## Impaired cerebral autoregulation

The mean arterial pressure (MAP) and intracranial pressure (ICP) determine the cerebral perfusion pressure (CPP) as CPP = MAP − ICP. The value of intracranial pressure is affected by cerebral blood flow (CBF) and cerebral blood volume (CBV) (Molnár et al. 2018; Goodson et al. 2018). Cerebral autoregulation (CAR) is a homeostatic mechanism that protects the brain tissue from the potentially damaging effects of hypo- and hyperperfusion (Crippa et al. 2018). Impaired autoregulation is one of the significant triggers of SAE pathogenesis (Ren et al. 2020). The diameter of cerebral arterioles also determines the severity of CAR disruption, as 40 to 200 μm in diameter of cerebral arterioles are said to be a significant contributor to both the autoregulatory and metabolic response of the brain circulation (Szatmári et al. 2010). Thus, their dilation beyond this range may result in a decrease in cerebrovascular resistance. High cerebrovascular resistance and disturbed cerebral autoregulation may expose septic patients to a decreased CBF if a compensatory elevation in CPP is absent (Molnár et al. 2018).

Altered CAR is an independent risk factor associated with SAE occurrence, while lower MAP, a history of chronic kidney disease, and fungal infection were associated with altered CAR (Crippa et al. 2018). One study determined the degree of CAR in a time-dependent manner. The authors reported that autoregulation was impaired in 60% of patients on day 1, 59% on day 2, 41% on day 3, and 46% on day 4; in addition, impaired autoregulation on day 1 was also associated with the presence of SAD on day 4 (Tsuruta and Oda 2016). Similarly, in a sheep model of SAE, the authors determined the time course of alterations of CAR and neurovascular coupling (NVC). They observed a progressive loss of dynamic CA (DCA) and NVC in septic shock associated with cortical neuronal dysfunction. This study indicated that the alteration of mechanisms controlling cortical perfusion is critical in the pathophysiology of SAE; hence, assessment of DCA and NVC in clinical practice is essential (Ferlini et al. 2020).

Loss of autoregulation is also implicated in brain oedema due to impaired CBF resulting in an altered microvascular system (Cotena and Piazza 2012). One study stated that cerebrovascular autoregulation depends on cerebral endothelial function, and endothelial dysfunction is a critical feature in sepsis (Pfister et al. 2008). Also, systemic inflammation triggers vascular dysfunction, which further disrupts CAR observed in SAE (Rivera-Lara 2019). Sustained cerebral dysfunction resulting from impaired CAR is potentially associated with reduced attention, disrupted sleep-wakefulness balance, impaired memory, speech, and orientation, focal neurological deficits and seizure activity, perception disorders, decreased consciousness and coma (Shulyatnikova and Verkhratsky 2020). Thus, a synergistic mechanism may be involved in cerebrovascular dysfunction and CAR impairment, leading to the pathogenesis of SAE. Both cerebrovascular dysfunction and microcirculatory changes result from impaired autoregulation leading to cerebral ischaemia and potentially SAE (Rosenblatt et al. 2020).

## Oxidative stress

Oxidative stress plays a significant role in inducing cell apoptosis and endothelial vasculopathy (Czempik et al. 2020; Lamar et al. 2011). An imbalance in oxidative stress disrupts cellular respiration and abnormal metabolism, producing free radicals, and further causing cell damage. Free radicals produced by these phenomena can induce inflammatory mediators and cause disruption of BBB and secondary brain damage (Vasiljevic et al. 2011). Activated glutamate caused increased production of ROS. ROS causes damage and oxidation of lipids, DNA, and proteins, leading to energy depletion. Calcium overload activates the release of nitric oxide synthase (NOS), leading to high levels of the toxic free radical neurotransmitter nitric oxide (NO). NO attack enzymes that are associated with oxidative phosphorylation and electron transfer. It also exacerbates brain damage by reducing neuronal energy production by inhibiting glycolytic and mitochondrial enzymes (Kostandy 2012), thereby increasing DNA damage.

Nox2, essential for glial cell activation, is the primary source of ROS in the oxidative damage to the hippocampus in SAE and Nox2-derived ROS is a determining factor for cognitive impairments after sepsis (Michels et al. 2015). Activated Nox2 was demonstrated by Hernandes et al. 2014 in a septic rat model using apocynin, an inhibitor of NADPH oxidative activity, which inhibited Nox2 and 4-HNE expressions in the hippocampus and prevented the development of long-term cognitive impairment in septic survivors (Hernandes et al. 2014). In another model, LPS induced increases in ROS generation, inducible nitric oxide synthase (iNOS) expression, and Nox production, as well as upregulation of G protein-coupled receptor kinase 2 (GRK2) cytosolic expression in LPS-stimulated microglia. The authors concluded that GRK2 is a critical regulator of cellular oxidative and nitrosative stress in LPS-stimulated microglia (Kawakami et al. 2018). An imbalance in neurotransmitters such as dopamine increases neuronal excitability. GABA and Ach, which decrease neuronal excitability, lead to neuronal instability and unpredictable neurotransmission (Tsuruta and Oda 2016), implicated in the SAE pathogenetic mechanism.

## Mitochondrial impairment

Mitochondria play a vital role in neuronal functions, and altering mitochondrial dynamics, including fission and fusion, can have deleterious effects. In an SAE model, the authors observed a decrease in cellular respiration and a shift towards glycolysis under LPS stimulation that further led to the loss of mitochondrial membrane potential, propagation of dynamin-related protein 1 (Drp1) and p53 recruitment to the mitochondrial outer membrane, with subsequent initiation of cell death pathways (Haileselassie et al. 2020). Increased ROS and NO result in decreased mitochondrial ATP generation, which induces neuronal apoptosis by releasing cytochrome C (Cotena and Piazza 2012). Reactive nitrogen species (RNS), NO and ROS activation inhibit complexes I and IV of the electron transport chain (ETC), disrupting mitochondrial function implicated in SAE pathogenesis. Furthermore, ROS/RNS enhance both endoplasmic reticulum and mitochondrial membrane permeability, which permits calcium and proapoptotic protein leakage into the cytoplasm (Nwafor et al. 2019; Heming et al. 2017). Exacerbated PICs activation lead to disruption of mitochondrial biogenesis. Zhao et al. 2017 assessed the expression of TNF-α, IL-6, ROS and NO at 0 h, 6 h, 12 h and 24 h time points in the neonatal SAE model and discovered that these factors were significantly increased in groups 6 h, 12 h and 24 h groups, resulting in ultrastructural damage of mitochondrial biogenesis (Zhao et al. 2017). In essence, there is a synergy between PICs upregulation and mitochondrial dysfunction involved in SAE pathogenesis. Knockdown of voltage-dependent anion channel 1 (VDAC1), a critical component of the mitochondrial permeability transition pore (MPTP), in a mouse model of SAE was shown to alleviate cognitive dysfunction secondary to SAE (Cai et al. 2021). This study highlighted the central role VDAC1 played in mitochondrial dysfunction during SAE pathogenesis.

## Immune cells

Immune cells are implicated in the pathogenesis of SAE, where monocyte/macrophage and microglial cells are activated, with subsequent infiltration of neutrophils associated with neuroinflammation. A 'vicious cycle' has been proposed as the primary mechanism involved in sepsis-induced immunosuppression leading to SAE pathogenesis. This vicious cycle involved the overactivation of neutrophils and CNS dysfunction caused by neutrophil infiltration (Ren et al. 2020). Recruitment of leukocytes (PMN) into the microcirculation leads to the interaction of adhesion molecules and selectins, which further dysregulates the immune response (Blom et al. 2015). Another potential mechanism by which immune cells participate in the SAE pathophysiological process is through meningeal CD4 + cells, which are an essential part of the inflammatory microenvironment related to CNS functions. Meninges function as a protective mechanism in the CNS; thus, damage or injury to this structure can be detrimental to the CNS. In an animal model of SAE, Luo et al. 2020 reported that LPS injection induced the activation of CD11b + monocyte/ macrophages in the peripheral blood and meninges, accompanied by the upregulation of meningeal PICs, as well as a decrease in the percentage of CD4 + T cells in the peripheral blood and meninges. They, therefore, concluded that reduced meningeal CD4 + T cells and related cytokine gene expression indicate the involvement of CD4 + T cells in the SAE induced by LPS injection. They also reported an increased upregulation of proBDNF, a precursor for mature brain-derived neurotrophic factor (BDNF), in circulating and meningeal immune cells. That upregulated proBDNF promotes the development of SAE via reducing peripheral CD4 + T cells and its infiltration into the meninges, further exacerbating the pathogenesis of SAE (Luo et al. 2020). Infiltration of regulatory T cells (Treg) and Th2 cells in the brain contribute to the attenuation of SAE and mental impairment alleviation in a mouse model by resolving neuroinflammation during the acute phase of sepsis (Saito et al. 2021).

## Apoptosis

Apoptosis is another mechanism involved in the pathogenesis of SAE, which may lead to cell death accompanied by autophagy. Extensive apoptosis of T cells, inhibited by the over-production of corticosteroids, has been associated with poor outcomes in sepsis-induced immunosuppression in a Fas/FasLdependent manner, which is one of the pathogenic mechanisms implicated in SAE occurrence (Ren et al. 2020). Neuronal sensitivity from increased levels of NO produced by activated microglia can exacerbate neuronal apoptosis (Nwafor et al. 2019). Chemokines also promote neuronal apoptosis, as evidenced by increased upregulation of Ccl2 or Cxcl2 protein levels in the LPS model, resulting in hippocampal neuron apoptosis, thus supporting a direct role of these chemokines in neuronal death (Wolff et al. 2009). Intraperitoneal hypertension (IAH) can also potentiate SAE occurrence by promoting neuronal apoptosis (He et al. 2018).

As mentioned earlier, numerous pathogenetic mechanisms have been postulated that underlie the pathogenesis of SAE, such as diffuse neuroaxonal injury and ischaemic brain injury, not only in animal models but also in postmortem and clinically admitted septic patients (Ehler et al. 2017). Variations in mtDNA makeup also play crucial roles in the development and protection from delirium during sepsis (Samuels et al. 2019). mtDNA makeup variant is also an area of consideration to examine whether mitochondrial DNA haplogroup dysfunction is a key risk factor in developing SAD/SAE pathogenesis.

### Clinical features

Manifestations of SAE include impaired consciousness, seizures, delirium, coma, focal cognitive deficits, hallucinations, abnormal sleep rhythms, personality changes, lack of concentration, and depressive symptoms (Nwafor et al. 2019; Ziaja 2013; Helbing et al. 2018). Other symptoms include confusion, disorientation, agitation, stupor and hypersomnolence (Zhang et al. 2012). Delirium, the most common feature of SAE, is associated with several adverse outcomes, including psychomotor activity, visual and functional memory, verbal fluency, and visual construction (Ziaja 2013). Sickness behaviour is also observed in most patients with SAE, characterised by fever, adaptive behavioural changes, and neuroimmune changes (Shulyatnikova and Verkhratsky 2020; Nwafor et al. 2019). Some paediatric patients may also present with new-onset refractory status epilepticus (NORSE) as an initial clinical sign, which is common in SAE patients (Huang et al. 2020).

### Clinical screening scales

Numerous assessment scales have been used to screen for depth of coma and delirium or predict treatment outcomes and prognostication in adults and children. Such scales include confusion assessment method (CAM), CAM-ICU, sequential organ failure assessment (SOFA), quick SOFA (qSOFA), Glasgow coma scale (GCS), assessment to intensive care environment (ATICE), Richmond agitation sedation scale (RASS), Full outline unresponsiveness (FOUR), acute physiology and chronic health evaluation Π (APACHE Π), paediatric risk of mortality assessment III (PRISM III), paediatric sequential organ failure assessment (pSOFA).

The pSOFA has recently been used to determine the number of organs involved and the severity of organ damage (Matics and Sanchez-Pinto 2017; Mohamed El-Mashad et al. 2020). FOUR is used in intubated patients, which is associated with high mortality (Heming et al. 2017). The FOUR scores are said to provide more incredible neurological details than GCS. It is superior to GCS due to the availability of brainstem reflexes and breathing patterns in children suspected of SAE (Wijdicks et al. 2005). As shortcomings, GCS cannot assess verbal scores or test brainstem reflexes in intubated patients. In addition to GCS, BSID Π and Bayley Π are often used in children suspected of sepsis or critical condition (Jenster et al. 2014) to assess disease severity, intervention and prognostic prediction. PRISM Ш is a mortality predictor for critically ill paediatric patients admitted to PICU (Kaur et al. 2020). Those used in adults include CAM-ICU (Ehler et al. 2017), SOFA or qSOFA (Yang et al. 2020), ATICE (Ziaja 2013) and the intensive care delirium screening checklist (ICDSC) (Tsuruta and Oda 2016). However, despite the availability of these screening tools, there is still controversy in their applicability (Chaudhry and Duggal 2014), especially in children, considering the delicacy and nature of their disease presentations and clinical manifestations.

### Animal models of SAE

The most commonly used animal models to induce SAE are caecal ligation and puncture (CLP), colon ascendens stent peritonitis (CASP), lipopolysaccharide (LPS), faecal slurry (FS), etc. The most commonly used models are LPS and CLP. Recent studies have outlined their pros and cons for a better understanding and interpretation of data obtained from these models (Savi et al. 2021). In general, the heterogeneity of patients with septic syndromes makes it challenging to replicate sepsis-type symptoms in animal models, such as preexisting conditions, age, genetic make-up, weight, sex, nutritional status, and aggravating factors like trauma. All these factors or conditions are sometimes clearly excluded in animal models before they are challenged with a single well-defined precipitating event (Moraes et al. 2021; Poli-de-Figueiredo et al. 2008). Because of these limitations, scoring systems have been developed to help validate some of these animal models for mimicking sepsis, such as the Murine Sepsis Score (MSS), which is reliable, sensitive and specific not only on CLP models but also on faecal slurry septic models (Shrum et al. 2014; Mai et al. 2018).

Though these models mimic sepsis/SAE and provide helpful information in understanding sepsis pathophysiological manifestations, they still need to catch up to the actual scenarios seen in human sepsis/SAE characteristics. Thus, such results obtained from these models, be they diagnostic, treatment mechanism(s) or outcome stratification, should be interpreted cautiously. In essence, mimicking the SAE model in paediatrics proves very challenging, including dosage of the potential treatment agent, assessment of physiological factors, depth of sedation in the SAE model and clinical presentation. Most of these physiological factors are difficult to initiate, imitate or assess because of the postnatally developmental changes. For instance, LPS injection can elicit an immune response violently by acting on TLR4dose-dependently (Luo et al. 2020). Also, the depth and duration of sedation are associated with poor behavioural outcomes (Kaur et al. 2016), thus, proving the limitations of these models in mimicking paediatric SAE. Therefore, the interpretation and accuracy of these models or their results become inconclusive, and translation into clinical practice becomes tricky. In addition, in most of the treatment strategies being proven or suggested through these models, translation into clinical practice still needs to be improved. As stated by Rittirsch et al. animal models of sepsis need to be redesigned to reflect more accurately the corresponding age of septic humans (Rittirsch et al. 2007), and therefore cautious interpretation and extrapolation of data obtained from these models into preclinical and clinical trials.

Indeed, animal models have provided insight into understanding sepsis's pathogenesis, but we still need to mimic the complete picture of sepsis encountered clinically. However, one cannot ignore the fact that animal models remain essential and play a crucial role in the development of new treatments and experimentation of emerging therapeutic agents for sepsis and its associated syndromes, as these models provide us with not only the basic understanding of pathophysiological and mechanistic processes of sepsis but also the basic information of pharmacologic and toxicology of a potential investigated drug because of their reproducibility and duplicability, which is impossible in humans.

### Management

#### Standard therapy

SAE treatment focuses on managing the underlying conditions, as there is no specific treatment protocol (Ziaja 2013). Antibiotics and supportive therapy are the mainstays of treatment, while the sedative medication is used to treat agitation features (Helbing et al. 2018). Judicious use of fluid therapy is also crucial. Control of organ dysfunction and metabolic alterations is also essential (Chung et al. 2020).

Fluid therapy is an integral part of the resuscitation protocol in septic patients to restore and maintain circulation, perfusion, adequate oxygen delivery, and normalising vital signs. However, these fluids pose a high risk of hyperchloremic metabolic acidosis, hyperkalemia, pathologic immune activation and cell damage, bleeding disorders, renal failure or life-threatening allergic responses as side effects (Gu et al. 2021) due to fluid overload accumulating into the microcirculatory system. These adverse effects remain insidious at the initial stage of fluid therapy but develop gradually, further exacerbating disease progression, especially in paediatric patients. However, a recent study has reported that cardiovascular collapse contributes most to excess death with rapid fluid resuscitation rather than fluid overload (Maitland et al. 2013). In addition, the effect of fluid resuscitation in children with severe illness has been questioned, especially in resource-limited settings (Maitland et al. 2011).

The mainstay of pharmacologic treatment is antibiotic administration started as soon as possible before or after obtaining appropriate cultures, which is a norm for suspected septic patients admitted to ICUs. However, antibiotic administration in children with sepsis is without risks, such as the increased risk of necrotising enterocolitis (NEC) and death, altered intestinal microbial colonisation, wheezing in infants, and increased BMI and incidence of obesity (Poggi and Dani 2018; Leonardi et al. 2019). Whereas SAE is not a direct infection, it becomes problematic to initiate antibiotic treatment because even recent sepsis guidelines do not mandate microbial therapy in systemic inflammatory response without infection to minimise the likelihood that those septic patients will become infected with the antimicrobial-resistant pathogen or will develop a drug-related adverse effect, as outlined in adults and paediatric sepsis guidelines (Coopersmith et al. 2018; Weiss et al. 2020). Interestingly, a quality improvement web-based calculator has been developed to help reduce the unnecessary use of antibiotics in children diagnosed with sepsis (Zayek et al. 2020).

In PICUs, about 90% of mechanically ventilated children receive sedatives as part of treatment. The practice of sedation is a clinical balance between both states of undersedation and oversedation, which represent hazards to the critically ill child. Undersedation may lead to distress and adverse events such as unintentional extubation or displacement of catheters and increased lengths of stay. Conversely, oversedation can cause cardiovascular depression and ileus, may interfere with comprehensive neurological examinations, and, with prolonged sedation, tolerance and withdrawal phenomena may occur. Opioids are the preferred analgesics because of their marked beneficial sedative effects. Combination with midazolam and benzodiazepines are often co-prescribed.

Nevertheless, opioids and benzodiazepines produce tolerance, dependence and several unwanted side effects, including cardiovascular and respiratory depression in children. Animal studies also suggest the risk of neurotoxicity and impaired neurodevelopment with these agents (Hayden et al. 2017). No specific sedative agent is recommended, but rather to avoid or discontinue them whenever possible (Mazeraud et al. 2020).

#### Agents approved for other indications

Drugs indicated for treating other disease entities are being explored in suspected SAE patients, with the controversy surrounding their effectiveness in adult animal models (Table 3). The most widely used medication is dexmedetomidine, which is neuroprotective by inhibiting neuronal apoptosis, reducing the sepsis-associated inflammatory response and improving BBB integrity, thus improving short-term mortality, more encephalopathy-free days, and shorter time on a ventilator (Czempik et al. 2020; Nwafor et al. 2019). A consecutive dexmedetomidine exposure (1 week) in the SAE model decreased neuronal apoptosis, enhanced cell viability in vitro and in vivo, and improved spatial and emotional dysfunction in CLP rats (Yin et al. 2019). A systematic review of animal and human studies about the effect of dexmedetomidine and clonidine on the inflammatory response in critical illness showed that α2 agonist drugs might potentially modify inflammatory and immune pathways in acute inflammatory conditions (Flanders et al. 2019). Though dexmedetomidine may effectively reduce ICU length of stay and time to extubation in critically ill ICU patients, there is an increased risk of bradycardia among patients treated with dexmedetomidine (Cruickshank et al. 2016). A Systematic Review on the efficacy of α2-agonists for sedation in Paediatric Critical Care reported inconclusive outcomes. In contrast, the authors showed that the reporting of study results using the outcome "time maintained at target sedation score' for clonidine or dexmedetomidine was poor (Hayden et al. 2017).

**Table 3:** Therapeutic agents in SAE treatment

Agent	Model	Mechanism	Refs.
Dexamethasone	CLP rats	Reduced inflammation in cerebral cortical cells; improved neurobehavioural function; reduced cortical cerebral oedema; increased autophagy by inhibiting mTOR signalling with SdDex severe cortical damage; induced neuronal apoptosis with HdDex	Zhou (2019)
Insulin	LPS rats	Inhibited inflammatory cytokines, oxidative stress in the cortex, hypothalamus and hippocampus improved brain tissue damage	Chen et al. (2014)
Metformin	CLP rats	Attenuated cognitive dysfunction	Tang et al. (2017)
		Decreased neuronal apoptosis	
		Increased anti-inflammatory factors and p-AKt	
Sevoflurane	CLP rats	Attenuated systemic inflammation	Bedirli et al. (2018)
		Reduced lipid peroxidation	
		Enhanced apoptotic genes expression	
Ecballium elaterium	CLP rats	Suppressed oxidant activity by decreasing TOS levels	Arslan et al. (2017)
		Anti-inflammatory effects by decreasing TNF-α expression level	
Melatonin	CLP mice	Increased survival tare improved neurobehavioural dysfunction by normalising BDNF and GDNF expressions in the hippocampus	Ji et al. (2018)
Erythropoietin	CLP rats	Altered oxidative parameters and energetic metabolism reversed cognitive impairment	Comim et al. (2012)
Resveratrol	CLP mice	Inhibited NLRP3/IL-1 axis	Sui et al. (2016)
		Reduced Iba and IL-1 expression levels	
		Improved spatial learning and memory capacity	
Hydrogen gas	CLP mice	Increased Nfr2 expression	Xie et al. (2020)
		Alleviated inflammatory cytokines, neuronal apoptosis and mitochondrial dysfunction	
Neuroglobin	CLP rats	Reduced neuronal apoptotic factors	Zhang et al. (2014)
		Improved histopathologic changes	
		Attenuated oxidative stress factors	
Myricitrin	CLP rats	Ameliorated neuroinflammation	Gong et al. (2019)
		Improved memory	
		Regulated NLRP3/Bax/Bcl signalling pathway	
Ethyl pyruvate	CLP mice	Inhibited NLRP3 inflammasome activation	Zhong (2020)
		Improved cognitive function	
		Decreased IL-1β release from microglia	
USP8	CLP mice	Attenuated cognitive and motor impairment	Bi (2019)
		Suppressed release of pro-inflammatory mediators	
Huperzine	LPS rats	Improved deficient cholinergic nervous function	Zhu (2016)
		Attenuated abnormal neuroinflammation	
Attractylone	LPS mice	Attenuated cognitive impairment, neural apoptosis, inflammatory	Tian (2019)
		factors, microglial activation	
		Promoted SIRT1 expression and suppressed NF-κB expression	
Ginsenoside Rg1	CLP mice	Attenuated brain histopathologic changes	Li (2017)
		Improved survival rate	
		Decreased inflammatory cytokine and reduced neuronal apoptosis	
Mdivi-1	LPS rats	Downregulated Drp1 level in a dose-dependent manner	Deng (2018)
		Attenuated brain damage, S100B and NSE release, and oxidative stress in a dose-dependent manner	
Butein	CLP mice	Prolonged survival rate; improved cognitive function; decreased cerebral oedema and maintained BBB integrity; decreased PICs production; increased activation of SIRT1 pathway	Zhu (2019)
FBP	Gelatin mice	Maintained and prevented glucose metabolism; reduced ROS release	Catarina (2018)
l-dopa	CLP, LPS mice	Improved neuroinflammation and cognitive function; attenuated neuroinflammation via D1 receptor; decreased hippocampal dopamine level; L-DA's protection on cognition was mediated by D1 and D2 receptors	Li (2020)
Bornoel	LPS mice	Attenuated brain neuronal and microglial inflammation; Suppressed the p-p65 and p38 pathways; blocked the activation of MAPK and NF-κB signalling pathways	Wang (2019)
Morin	CLP mice	Downregulated the expressions of IL-6, MCP-1, TNF-α and IL-10; Diminished microglia activation; reduced phosphokinase GSK3β and elevated phosphatase PP2A activity; reduced Aβ deposition and protected synaptic integrity	Xu et al. (2020)
Fisetin	CLP rats	Blocked NLRP3 inflammasome activation via mitophagy; reduced neuroinflammation; ameliorated cognitive impairment	Ding (2022)
KYNA	F S rats	Reduced peripheral NET formation; lowered BBB permeability changes; alleviated mitochondrial dysfunction	Poles (2021)

*Bax* Bcl-2 associated X protein, *Bcl-2* B-cell lymphoma 2, *BDNF* Brain-derived neurotrophic factor, *CLP* Caecal ligation and puncture, *GDNF* Glial-derived neurotrophic factor, *HdDex* High dose dexamethasone, *IL-1β* Interleukin-1 beta, *LPS* Lipopolysaccharide, *mTOR* mammalian target of rapamycin, *NF-κB* Nuclear factor-kappa B, *Nfr2* Nuclear factor erytheroid 2-related factor 2, *NLRP3* Nucleotide-binding domain-like receptor factor 3, *p-Akt *Phosphorylated protein kinase B, *SdDex* Small dose dexamethasone, *SIRT1* Silent information regulator 1, *TNF-α *Tumour necrosis factor-alpha, *TOS* Total oxidant status, *USP8 *Ubiquitin-specific protease 8, *KYNA* Kynurenic acid, *NET* Neutrophil extracellular trap, *MCP-1* Monocyte chemoattractant protein 1, *PP2A* Protein phosphatase 2A, *BBB* Blood–brain barrier, *FBP* Fructose-1, 6-bisphosphate, *ERK* Extracellular signal regulated kinase, *GSK-3β* Glucogen synthase kinase-3 beta, *MAPK* Mitogen-activated protein kinase, *NSE* Neuron specific enolase, *SAE* Sepsis associated encephalopathy, *FS* Faecal slurry

A prospective, randomised, controlled trial has shown that plasmapheresis may reduce mortality in patients with severe sepsis or septic shock. However, this study failed to make general recommendations due to few data availability during the study period (Busund et al. 2002). A recent case report showed that therapeutic plasma exchange (TPE) effectively eliminates pro-inflammatory cytokines and modulates sepsis cascade in a 5-year-old child with fulminant encephalopathy complicated by hyperferritinemia sepsis (Huang et al. 2020). TPE is a non-selective intervention that removes multiple toxic mediators, including endotoxins, PICs and procoagulant factors (Hadem et al. 2014).

Dexamethasone is an effective agent in SAE subjects. Zhou et al. 2019 compared low and high doses of dexamethasone in a juvenile SAE model and reported that a low dose of dexamethasone significantly increased blood levels of IL-10, reduced levels of TNF-a and lowered bacterial blood load, as well as increased autophagy in cerebral cortical neurons by inhibiting the mTOR signalling pathway. In contrast, high-dose dexamethasone resulted in severe cortical damage, no improvement in cerebral oedema, and disordered neuronal structure via the activation of caspase-3 (Zhou et al. 2019). In infants diagnosed with SAE complicated by biliary atresia, dexamethasone improved infection severity and overall neurological outcome (Abe et al. 2008).

Melatonin regulates the circadian rhythm and also has anti-inflammatory and antioxidant properties. In a study by Ji et al., the authors examined melatonin's short- and long-term effects in a mouse model of SAE. They observed that early melatonin treatment increased survival rate and decreased IL-1β, while delayed melatonin administration improved neurobehavioural dysfunction by normalising hippocampal BDNF and GDNF expression levels (Ji et al. 2018). Insulin is also a potential candidate for treating subjects with SAE by suppressing oxidative stress, ameliorating mitochondrial function or inhibiting the release of cytokines in septic patients and animals (Chen et al. 2014).

Despite the controversy about the beneficial effect of statins in treating sepsis, a comparable number of studies have shown statins to reduce glial activation, regulate mitochondrial bioenergetics, restore balance in redox reactions, and reduce microvascular damage and apoptosis (Reis et al. 2017; Catalão et al. 2020). Ketamine is potentially neuroprotective in SAE but has not yet been in interventional trials (Mazeraud et al. 2020). Intravenous immunoglobulin (IVIG) binds to Fc receptors (FcγRs) which neutralise endotoxins/cytokines, inhibit complement activation, and block leukocyte adhesion molecule binding. It is also effective in septic patients (Takemoto et al. 2019; Nwafor et al. 2019).

Molecular hydrogen (H2) is said to exert its antineuroinflammatory effects associated with TLR4/NF-κb activation and neuroprotective effects by inhibiting the excessive release of PICs and neuronal loss and apoptosis via the Nrf2 signalling pathway (Xie et al. 2020; Chen et al. 2021). Inhaled sevoflurane exerts its neuroprotective effects in the SAE rat model by enhancing the expression of apoptotic genes as well as decreasing memory impairment (Bedirli et al. 2018). The effect of sevoflurane on mortality and inflammatory parameters is currently being assessed (NCT03643367). The neuroprotective effect of isoflurane is through the activation of HO-1, which mediates anti-inflammatory, antioxidative and anti-apoptotic effects in the SAE model (Zhang et al. 2021). Metformin (Tang et al. 2017; Ismail Hassan et al. 2020), EPO (Comim et al. 2012; Gao et al. 2015), L-dopa/benserazide (Li et al. 2020), Ethyl pyruvate (Zhong et al. 2020), Neuroglobin (Ngb) (Zhang et al. 2014), Ubiquitin-specific protease 8 (USP8) (Bi et al. 2019), Ecballium elaterium (EE) (Arslan et al. 2017), Myricitrin (Gong et al. 2019), Electroacupuncture (EA) (Li et al. 2020; Mo et al. 2021), Resveratrol (Sui et al. 2016), Attractylone (Tian et al. 2019), Ginsenoside (Li et al. 2017), Mdivi-1 (Deng et al. 2018), Butein (Zhu et al. 2019), Boenoel (Wang et al. 2019), Morin (Xu et al. 2020), Fisetin (Ding et al. 2022), Kynurenic acid (Poles et al. 2021), etc. all are neuroprotective in SAE adult models. However, some of these agents, in some ways, have failed a successful translation from bench to bedside (Flierl et al. 2010). Therefore more research is needed to evaluate and validate their effectiveness, especially in children.

Maintaining intestinal microbiota integrity is very crucial in patients with SAE. Faecal microbiota transplantation (FMT) is one of the agents implicated in maintaining this integrity (Li et al. 2018). A recent study compared the efficacy of 4 therapeutic methods to modify gut microbiota dysbiosis and brain dysfunction in septic rats exposed to LPS, i.e. FMT, prebiotics, probiotics, and synbiotics. FMT was the most effective method for correcting dysbiosis and restoring the normal gut microflora (Li et al. 2021).

Short-chain fatty acids (SCFAs), produced by gut microflora metabolising dietary fibre, are shown to improve abnormal behaviour, neuronal degeneration, and BBB impairment in the SAE mice, to decrease excessive activation of microglia and production of pro-inflammatory cytokines, such as IL-1β and IL-6, to increase the expression levels of tight junction-associated proteins, such as Occludin and ZO-1, and decrease the phosphorylation levels of JNK and NF-kB p65 in the brain of SAE mice (Liu et al. 2021). In contrast, in the septic mice model, fructose-1, 6-bisphosphate (FBP) was shown to maintain and prevent glucose metabolism and reduce ROS release (Catarina et al. 2018).

Managing modifiable factors associated with SAE is also crucial, such as hypoglycaemia, hyperglycaemia, hypercapnia, and hypernatremia (Czempik et al. 2020). Non-pharmacological approaches are also necessary to prevent and manage delirium as an acute symptom of SAE; this includes but is not limited to reorientation, anxiety reduction and general measures such as reinforcement of regular circadian sleep cycles, early mobilisation, occupational therapy and physiotherapy, encouraging mental activity, music therapy, and ensuring sufficient nutrients and fluid intake (Tauber et al. 2021; Chung et al. 2020).

## Perspective and conclusion

SAE is one of the most common types of sepsis-related organ dysfunction associated with high mortality, lower quality of life and long-term neurological sequelae. It has gained much attention from clinicians and researchers because of these neurological consequences, and its prevalence remains uncertain. The pathophysiology of SAE is multifactorial, involving diffuse neuroinflammation, disrupted BBB, mitochondrial dysfunction, oxidative stress, excitotoxicity and cerebral autoregulation impairment. Early diagnosis of SAE is crucial for appropriate intervention protocols, such as EEG, SEP, MRI and biomarker detection, to guide treatment regimens, treatment effects, prognostic evaluation and anticipated neurological outcomes. However, SAE remains a diagnostic of exclusion wherein other encephalopathies with related characteristics are diagnosed first, posing a delay in the anticipated timely intervention.

Interestingly, some gaps and puzzles need to be solved. For instance, the consistency and validity of these models used to induce sepsis, the efficacy of investigated drugs, and the lack of a paediatric animal model of sepsis. Key issues should also be taken into consideration when interpreting and extrapolating animal models clinically, including physiological differences between animals and humans in response to infections; consistency in reproducibility of findings; involvement of peripheral organs when inducing septic models to a specific organ, and technical consistency in manipulating these models and interpreting of findings (Moraes et al. 2021). Thus, the standardisation of animal models while solving these key differences is worth considering. In addition, there is no single study investigating any potential drug in a paediatric septic model, which is alarming considering the prevalence of sepsis in children and its associated mortality and long-term neurodevelopmental sequelae.

Furthermore, recent clinical trials are yet to show any efficacy of these new treatment strategies that are effective in preclinical animal models. Moreover, one of the contributing factors is the misinterpretation of preclinical data obtained from animal experimentations because these models need to adequately mimic human sepsis with its clinical manifestations (Poli-de-Figueiredo et al. 2008). The efficacious effects of these agents need validation in extensive clinical studies.

Though the pathophysiology of SAE is being explored with limited treatment options other than systemic support and antibiotics that are sometimes associated with brain dysfunction in critically ill patients as side effects, especially in children due to the complexity of their brain development and disease course, at the moment, it is noteworthy that judicious use of empirical regimen is the mainstay of managing SAE patients. Even those emerging agents under investigation are mainly focused on SAE adult models, and clinical trials are needed to investigate their efficacy.

This review highlighted the current understanding of SAE pathogenetic mechanisms, diagnostic paradigms and treatment strategies. It is also noteworthy that the long-term mortality and sequelae associated with SAE are more pronounced in children and thus pose high economic, social and parental burdens. Therefore, it is prudent to prioritise early diagnostic and interventional strategies to mitigate its short- and long-term neurological consequences in paediatric patients. Numerous models mimicking SAE pathogenesis and mode of action have expounded our understanding of its mode of action, possible management strategies, and potential emerging agents.

## Data Availability

Not applicable.

## Abbreviations

AAA: Aromatic amino acids
ADC: Apparent diffusion coefficient
AKt: Protein kinase B
APACHEΠ: Acute physiology and chronic health evaluation Π
ATICE: Assessment to intensive care environment
BBB: Blood brain barrier
BCAA: Branch chain amino acids
BCSFB: Blood cerebrospinal fluid barrier
BDNF: Brain-derived neurotrophic factor
BMVELs: Brain microvascular endothelial cells
BSIDΠ: Bayley scales of neonatal development
CAP: Cholinergic anti-inflammatory pathway
CAM: Confusion assessment method
CAR: Cerebral autoregulation
CASP: Colon ascendens stent peritonitis
CBF: Cerebral blood flow
CBV: Cerebral blood volume
CLP: Caecal ligation and puncture
CNS: Central nervous system
CPP: Cerebral perfusion pressure
CRC: Cerebrovascular reserve capacity
CREB: CAMP responsive element binding-protein
CVR: Cerebrovascular resistance
DWI: Diffusion weighted imaging
EEG: Electroencephalograph
EPO: Erythropoietin
ERK1/2: Extracellular signal-regulated kinase 1/2
FLAIR: Fluid attenuated inversion recovery
FMT: Faecal microbiota transplantation
FOUR: Full outline unresponsiveness
GCS: Glasgow coma scale
GDNF: Growth-derived neurotrophic factor
GFAP: Glial fibrillary acidic protein
GRK2: G protein-coupled receptor kinase 2
GSH: Glutathione
HPA: Hypothalamic pituitary adrenal axis
ICAM-1: Intercellular adhesion molecule-1
ICH: Intracranial hypertension
IKKB: Inhibitor of kappa B kinase
iNOS: Inducible nitric oxide synthase
JAK2: Janus kinase 2
LPS: Lipopolysaccharide
MAP: Mean arterial pressure
MCP1: Monocyte chemoattractant protein 1
MMP: Matrix metalloproteinase
MRI: Magnetic resonance imaging
mTOR: Mammalian target of rapamycin
NF-κB: Nuclear factor-kappa B
NLRP3: Nucleotide-binding domain-like receptor protein 3
NMDARs: N-methyl-D-aspartate receptors
NO: Nitric oxide
NSE: Neuron specific enolase
PaCO2: Arterial partial pressure of carbon dioxide
PI3K: Phosphatidylinositol 3-kinase
PICs: Pro-inflammatory cytokines
PICU: Paediatric intensive care unit
PRISMШ: Paediatric risk of mortality assessment
PSD95: Postsynaptic density protein 95
pSOFA: Paediatric sequential organ failure assessment
qSOFA: Quick sequential organ failure assessment
RASS: Richmond agitation sedation scale
RDW: Red blood cell distribution width
RNS: Reactive nitrogen species
ROS: Reactive oxygen species
rSCO2: Regional cerebral oxygen saturation
SAE: Sepsis-associated encephalopathy
SABD: Sepsis-associated brain dysfunction
SAD: Sepsis-associated delirium
SCVO2: Central venous oxygen saturation
SEPs: Sensory evoked potentials
SIBD: Sepsis-induced brain dysfunction
SOD: Superoxide dismutase
SOFA: Sequential organ failure assessment
SPECT: Single positron emission computerised topography
STAT3: Signal transducer and activated protein kinase 3
STEP: Striatal-enriched protein tyrosine phosphatase
TCD: Transcranial doppler
TGF-β1: Transforming growth factor beta 1
TLR4: Toll-like receptor 4
TNF-α: Tumour necrosis factor alpha
TNFR: Tumour necrosis factor receptor
TOS: Total oxidant status
TPE: Therapeutic plasma exchange
VCAM-1: Vascular cell adhesion molecule-1
VMR: Vasomotor reactivity

## References

[CR1] Abe S, Okumura A, Fujii T, Someya T, Tadokoro R, Arai Y, Nakazawa T, Yamashiro Y (2008). Sepsis associated encephalopathy in an infant with biliary atresia. Brain Dev.

[CR2] Alexander JJ, Jacob A, Cunningham P, Hensley L, Quigg RJ (2008). TNF is a key mediator of septic encephalopathy acting through its receptor, TNF receptor-1. Neurochem Int..

[CR3] Algebaly H, ElSherbini S, Galal A, Hamdi R, Baz A, Elbeleidy A (2020). Transcranial Doppler can predict development and outcome of sepsis-associated encephalopathy in pediatrics with severe sepsis or septic shock. Front Pediatr.

[CR4] Andonegui G, Zelinski EL, Schubert CL, Knight D, Craig LA, Winston BW, Spanswick SC, Petri B, Jenne CN, Sutherland JC, Nguyen R, Jayawardena N, Kelly MM, Doig CJ, Sutherland RJ, Kubes P (2018). Targeting inflammatory monocytes in sepsis-associated encephalopathy and long-term cognitive impairment. JCI Insight..

[CR5] Archie SR, Al Shoyaib A, Cucullo L (2021). Blood-brain barrier dysfunction in CNS disorders and putative therapeutic targets: an overview. Pharmaceutics.

[CR6] Arslan D, Ekinci A, Arici A, Bozdemir E, Akil E, Ozdemir HH (2017). Effects of Ecballium elaterium on brain in a rat model of sepsis-associated encephalopathy. Libyan J Med.

[CR7] Banks WA, Gray AM, Erickson MA, Salameh TS, Damodarasamy M, Sheibani N, Meabon JS, Wing EE, Morofuji Y, Cook DG, Reed MJ (2015). Lipopolysaccharide-induced blood–brain barrier disruption: roles of cyclooxygenase, oxidative stress, neuroinflammation, and elements of the neurovascular unit. J Neuroinflamm.

[CR8] Barbosa-Silva MC, Lima MN, Battaglini D, Robba C, Pelosi P, Rocco PRM, Maron-Gutierrez T (2021). Infectious disease-associated encephalopathies. Crit Care.

[CR9] Bartha AI, Foster-Barber A, Miller SP, Vigneron DB, Glidden DV, Barkovich AJ, Ferriero DM (2004). Neonatal encephalopathy: association of cytokines with MR spectroscopy and outcome. Pediatr Res.

[CR10] Becker AE, Teixeira SR, Lunig NA, Mondal A, Fitzgerald JC, Topjian AA, Weiss SL, Griffis H, Schramm SE, Traynor DM, Vossough A, Kirschen MP (2021). Sepsis-related brain MRI abnormalities are associated with mortality and poor neurological outcome in pediatric sepsis. Pediatr Neurol..

[CR11] Bedirli N, Bagriacik EU, Yilmaz G, Ozkose Z, Kavutçu M, CavuntBayraktar A, Bedirli A (2018). Sevoflurane exerts brain-protective effects against sepsis-associated encephalopathy and memory impairment through caspase 3/9 and Bax/Bcl signaling pathway in a rat model of sepsis. J Int Med Res.

[CR12] Bi W, Lan X, Zhang J, Xiao S, Cheng X, Wang H, Lu D, Zhu L (2019). USP8 ameliorates cognitive and motor impairments via microglial inhibition in a mouse model of sepsis-associated encephalopathy. Brain Res.

[CR13] Blom C, Deller BL, Fraser DD, Patterson EK, Martin CM, Young B, Liaw PC, Yazdan-Ashoori P, Ortiz A, Webb B, Kilmer G, Carter DE, Cepinskas G (2015). Human severe sepsis cytokine mixture increases β2-integrin-dependent polymorphonuclear leukocyte adhesion to cerebral microvascular endothelial cells in vitro. Crit Care.

[CR14] Bozza FA, Garteiser P, Oliveira MF, Doblas S, Cranford R, Saunders D, Jones I, Towner RA, Castro-Faria-Neto HC (2010). Sepsis-associated encephalopathy: a magnetic resonance imaging and spectroscopy study. J Cereb Blood Flow Metab..

[CR15] Brochu ME, Girard S, Lavoie K, Sébire G (2011). Developmental regulation of the neuroinflammatory responses to LPS and/or hypoxia-ischemia between preterm and term neonates: an experimental study. J Neuroinflammation.

[CR16] Busund R, Koukline V, Utrobin U, Nedashkovsky E (2002). Plasmapheresis in severe sepsis and septic shock: a prospective, randomised, controlled trial. Intensive Care Med.

[CR17] Cai M, Du B, Si Y, Miao J, Ge J, Zhang J, Song J, Bao H (2021). Knockdown of VDAC1 alleviates the cognitive dysfunction secondary to sepsis-associated encephalopathy. Am J Transl Res..

[CR18] Catalão CHR, Santos-Junior NN, da Costa LHA, Souza AO, Cárnio EC, Sebollela A, Alberici LC, Rocha MJA (2020). Simvastatin Prevents long-term cognitive deficits in sepsis survivor rats by reducing neuroinflammation and neurodegeneration. Neurotox Res.

[CR19] Catarina AV, Luft C, Greggio S, Venturin GT, Ferreira F, Marques EP, Rodrigues L, Wartchow K, Leite MC, Gonçalves CA, Wyse ATS, Da Costa JC, De Oliveira JR, Branchini G, Nunes FB (2018). Fructose-1,6-bisphosphate preserves glucose metabolism integrity and reduces reactive oxygen species in the brain during experimental sepsis. Brain Res.

[CR20] Catarina AV, Branchini G, Bettoni L, De Oliveira JR, Nunes FB (2021). Sepsis-associated encephalopathy: from pathophysiology to progress in experimental studies. Mol Neurobiol.

[CR21] Chacqueneau AL, Desrumaux-Becquet A, Debillon T, NGuyen MA, Bessaguet S, Bost-Bru C, Leroy P, Wroblewski I (2013). Encéphalopathie associée au sepsis (EAS), un cas pédiatrique [A child with sepsis-associated encephalopathy]. Arch Pediatr..

[CR22] Chaudhry N, Duggal AK (2014). Sepsis associated encephalopathy. Adv Med.

[CR23] Chen Q, Yu W, Shi J, Shen J, Gao T, Zhang J, Xi F, Li J, Li N (2014). Insulin alleviates the inflammatory response and oxidative stress injury in cerebral tissues in septic rats. J Inflamm (lond).

[CR24] Chen J, Shi X, Diao M, Jin G, Zhu Y, Hu W, Xi S (2020). A retrospective study of sepsis-associated encephalopathy: epidemiology, clinical features and adverse outcomes. BMC Emerg Med.

[CR25] Chen H, Dong B, Shi Y, Yu Y, Xie K (2021). Hydrogen alleviates neuronal injury and neuroinflammation induced by microglial activation via the nuclear factor erythroid 2-related factor 2 pathway in sepsis-associated encephalopathy. Neuroscience.

[CR26] Chung HY, Wickel J, Brunkhorst FM, Geis C (2020). Sepsis-associated encephalopathy: from delirium to dementia?. J Clin Med.

[CR27] Comim CM, Cassol OJ, Abreu I, Moraz T, Constantino LS, Vuolo F, Galant LS, de Rochi N, Dos Santos Morais MO, Scaini G, Barichello T, Streck EL, Quevedo J, Dal-Pizzol F (2012). Erythropoietin reverts cognitive impairment and alters the oxidative parameters and energetic metabolism in sepsis animal model. J Neural Transm (vienna).

[CR28] Coopersmith CM, De Backer D, Deutschman CS, Ferrer R, Lat I, Machado FR, Martin GS, Martin-Loeches I, Nunnally ME, Antonelli M, Evans LE, Hellman J, Jog S, Kesecioglu J, Levy MM, Rhodes A (2018). Surviving sepsis campaign: research priorities for sepsis and septic shock. Intensive Care Med.

[CR29] Cotena S, Piazza O (2012). Sepsis-associated encephalopathy. Transl Med UniSa..

[CR30] Crippa IA, Subirà C, Vincent JL, Fernandez RF, Hernandez SC, Cavicchi FZ, Creteur J, Taccone FS (2018). Impaired cerebral autoregulation is associated with brain dysfunction in patients with sepsis. Crit Care.

[CR31] Cruickshank M, Henderson L, MacLennan G, Fraser C, Campbell M, Blackwood B, Gordon A, Brazzelli M (2016). Alpha-2 agonists for sedation of mechanically ventilated adults in intensive care units: a systematic review. Health Technol Assess.

[CR32] Czempik PF, Pluta MP, Krzych ŁJ (2020). Sepsis-associated brain dysfunction: a review of current literature. Int J Environ Res Public Health.

[CR33] Czempik PF, Gąsiorek J, Bąk A, Krzych ŁJ (2020). Ultrasonic assessment of optic nerve sheath diameter in patients at risk of sepsis-associated brain dysfunction: a preliminary report. Int J Environ Res Public Health.

[CR34] Deng S, Ai Y, Gong H, Feng Q, Li X, Chen C, Liu Z, Wang Y, Peng Q, Zhang L (2018). Mitochondrial dynamics and protective effects of a mitochondrial division inhibitor, Mdivi-1, in lipopolysaccharide-induced brain damage. Biochem Biophys Res Commun.

[CR35] Ding H, Li Y, Chen S, Wen Y, Zhang S, Luo E, Li X, Zhong W, Zeng H (2022). Fisetin ameliorates cognitive impairment by activating mitophagy and suppressing neuroinflammation in rats with sepsis-associated encephalopathy. CNS Neurosci Ther..

[CR36] Ebersoldt M, Sharshar T, Annane D (2007). Sepsis-associated delirium. Intensive Care Med.

[CR37] Ehler J, Barrett LK, Taylor V, Groves M, Scaravilli F, Wittstock M, Kolbaske S, Grossmann A, Henschel J, Gloger M, Sharshar T, Chretien F, Gray F, Nöldge-Schomburg G, Singer M, Sauer M, Petzold A (2017). Translational evidence for two distinct patterns of neuroaxonal injury in sepsis: a longitudinal, prospective translational study. Crit Care.

[CR38] Ehler J, Petzold A, Wittstock M, Kolbaske S, Gloger M, Henschel J, Heslegrave A, Zetterberg H, Lunn MP, Rommer PS, Grossmann A, Sharshar T, Richter G, Nöldge-Schomburg G, Sauer M (2019). The prognostic value of neurofilament levels in patients with sepsis-associated encephalopathy—a prospective, pilot observational study. PLoS ONE.

[CR39] El Shimy MS, El-Raggal NM, El-Farrash RA, Shaaban HA, Mohamed HE, Barakat NM, Farag AS, El Zohiery AK, Shaaban MAA, Salama DH (2018). Cerebral blood flow and serum neuron-specific enolase in early-onset neonatal sepsis. Pediatr Res.

[CR40] Ferlini L, Su F, Creteur J, Taccone FS, Gaspard N (2020). Cerebral autoregulation and neurovascular coupling are progressively impaired during septic shock: an experimental study. Intensive Care Med Exp.

[CR41] Flanders CA, Rocke AS, Edwardson SA, Baillie JK, Walsh TS (2019). The effect of dexmedetomidine and clonidine on the inflammatory response in critical illness: a systematic review of animal and human studies. Crit Care.

[CR42] Flierl MA, Rittirsch D, Huber-Lang MS, Stahel PF (2010). Pathophysiology of septic encephalopathy—an unsolved puzzle. Crit Care.

[CR43] Gao R, Tang YH, Tong JH, Yang JJ, Ji MH, Zhu SH (2015). Systemic lipopolysaccharide administration-induced cognitive impairments are reversed by erythropoietin treatment in mice. Inflammation.

[CR44] Gong J, Luo S, Zhao S, Yin S, Li X, Mou T (2019). Myricitrin attenuates memory impairment in a rat model of sepsis-associated encephalopathy via the NLRP3/Bax/Bcl pathway. Folia Neuropathol.

[CR45] Goodson CM, Rosenblatt K, Rivera-Lara L, Nyquist P, Hogue CW (2018). Cerebral blood flow autoregulation in sepsis for the intensivist: why its monitoring may be the future of individualized care. J Intensive Care Med.

[CR46] Griton M, Dhaya I, Nicolas R, Raffard G, Periot O, Hiba B, Konsman JP (2020). Experimental sepsis-associated encephalopathy is accompanied by altered cerebral blood perfusion and water diffusion and related to changes in cyclooxygenase-2 expression and glial cell morphology but not to blood-brain barrier breakdown. Brain Behav Immun.

[CR47] Gu M, Mei XL, Zhao YN (2021). Sepsis and cerebral dysfunction: BBB damage, neuroinflammation, oxidative stress, apoptosis and autophagy as key mediators and the potential therapeutic approaches. Neurotox Res.

[CR48] Guo J, Cheng Y, Wang Q, Su J, Cui L, Jin Z (2019). Changes of rScO2 and ScvO2 in children with sepsis-related encephalopathy with different prognoses and clinical features. Exp Ther Med..

[CR49] Guo W, Li Y, Li Q (2021). Relationship between miR-29a levels in the peripheral blood and sepsis-related encephalopathy. Am J Transl Res..

[CR50] Hadem J, Hafer C, Schneider AS, Wiesner O, Beutel G, Fuehner T, Welte T, Hoeper MM, Kielstein JT (2014). Therapeutic plasma exchange as rescue therapy in severe sepsis and septic shock: retrospective observational single-centre study of 23 patients. BMC Anesthesiol.

[CR51] Haileselassie B, Joshi AU, Minhas PS, Mukherjee R, Andreasson KI, Mochly-Rosen D (2020). Mitochondrial dysfunction mediated through dynamin-related protein 1 (Drp1) propagates impairment in blood brain barrier in septic encephalopathy. J Neuroinflammation.

[CR52] Hamasaki MY, Severino P, Puga RD, Koike MK, Hernandes C, Barbeiro HV, Barbeiro DF, Machado MCC, Reis EM, PinheirodaSilva F (2019). Short-term effects of sepsis and the impact of aging on the transcriptional profile of different brain regions. Inflammation.

[CR53] Hamed SA, Hamed EA, Zakary MM (2009). Oxidative stress and S-100B protein in children with bacterial meningitis. BMC Neurol.

[CR54] Hayden JC, Dawkins I, Breatnach C, Leacy FP, Foxton J, Healy M, Cousins G, Gallagher PJ, Doherty DR (2017). Effectiveness of α_2_agonists for sedation in paediatric critical care: study protocol for a retrospective cohort observational study. BMJ Open.

[CR55] He YJ, Xu H, Fu YJ, Lin JY, Zhang MW (2018). Intraperitoneal hypertension, a novel risk factor for sepsis-associated encephalopathy in sepsis mice. Sci Rep.

[CR56] Helbing DL, Böhm L, Witte OW (2018). Sepsis-associated encephalopathy. CMAJ.

[CR57] Heming N, Mazeraud A, Verdonk F, Bozza FA, Chrétien F, Sharshar T (2017). Neuroanatomy of sepsis-associated encephalopathy. Crit Care.

[CR58] Hernandes MS, D'Avila JC, Trevelin SC, Reis PA, Kinjo ER, Lopes LR, Castro-Faria-Neto HC, Cunha FQ, Britto LR, Bozza FA (2014). The role of Nox2-derived ROS in the development of cognitive impairment after sepsis. J Neuroinflamm.

[CR59] Hosokawa K, Gaspard N, Su F, Oddo M, Vincent JL, Taccone FS (2014). Clinical neurophysiological assessment of sepsis-associated brain dysfunction: a systematic review. Crit Care.

[CR60] Huang L, Peng S, Li R, Xie D, Huang D (2020). Fulminant encephalopathy in a child with hyperferritinemic sepsis: a case report. BMC Neurol.

[CR61] Ismail Hassan F, Didari T, Baeeri M, Gholami M, Haghi-Aminjan H, Khalid M, Navaei-Nigjeh M, Rahimifard M, Solgi S, Abdollahi M, Mojtahedzadeh M (2020). Metformin attenuates brain injury by inhibiting inflammation and regulating tight junction proteins in septic rats. Cell J.

[CR62] James A, Patel V. Hypoxic ischaemic encephalopathy. In: Symposium: Neonatology 2014, Paediatrics and Child Health 24:9.

[CR63] Jenster M, Bonifacio SL, Ruel T, Rogers EE, Tam EW, Partridge JC, Barkovich AJ, Ferriero DM, Glass HC (2014). Maternal or neonatal infection: association with neonatal encephalopathy outcomes. Pediatr Res..

[CR64] Ji MH, Xia DG, Zhu LY, Zhu X, Zhou XY, Xia JY, Yang JJ (2018). Short- and long-term protective effects of melatonin in a mouse model of sepsis-associated encephalopathy. Inflammation.

[CR65] Kaur J, Singhi P, Singhi S, Malhi P, Saini AG (2016). Neurodevelopmental and behavioral outcomes in children with sepsis-associated encephalopathy admitted to pediatric intensive care unit: a prospective case control study. J Child Neurol.

[CR66] Kaur A, Kaur G, Dhir SK, Rai S, Sethi A, Brar A, Singh P (2020). Pediatric risk of mortality III score—predictor of mortality and hospital stay in pediatric intensive care unit. J Emerg Trauma Shock..

[CR67] Kawakami M, Hattori M, Ohashi W, Fujimori T, Hattori K, Takebe M, Tomita K, Yokoo H, Matsuda N, Yamazaki M, Hattori Y (2018). Role of G protein-coupled receptor kinase 2 in oxidative and nitrosative stress-related neurohistopathological changes in a mouse model of sepsis-associated encephalopathy. J Neurochem.

[CR68] Kodali MC, Chen H, Liao FF (2021). Temporal unsnarling of brain's acute neuroinflammatory transcriptional profiles reveals panendothelitis as the earliest event preceding microgliosis. Mol Psychiatry.

[CR69] Kondo A, Sugiura C, Fujii Y, Inoue T, Maegaki Y, Ohno K (2009). Fulminant sepsis-associated encephalopathy in two children: serial neuroimaging findings and clinical course. Neuropediatrics.

[CR70] Kostandy BB (2012). The role of glutamate in neuronal ischemic injury: the role of spark in fire. Neurol Sci.

[CR71] Kuperberg SJ, Wadgaonkar R (2017). Sepsis-associated encephalopathy: the blood-brain barrier and the sphingolipid rheostat. Front Immunol.

[CR72] Lamar CD, Hurley RA, Taber KH (2011). Sepsis-associated encephalopathy: review of the neuropsychiatric manifestations and cognitive outcome. J Neuropsychiatry Clin Neurosci..

[CR73] Lang GP, Ndongson-Dongmo B, Lajqi T, Brodhun M, Han Y, Wetzker R, Frasch MG, Bauer R (2020). Impact of ambient temperature on inflammation-induced encephalopathy in endotoxemic mice-role of phosphoinositide 3-kinase gamma. J Neuroinflamm.

[CR74] Leonardi BM, Binder M, Griswold KJ, Yalcinkaya GF, Walsh MC (2019). Utilization of a neonatal early-onset sepsis calculator to guide initial newborn management. Pediatr Qual Saf..

[CR75] Li Y, Wang F, Luo Y (2017). Ginsenoside Rg1 protects against sepsis-associated encephalopathy through beclin 1-independent autophagy in mice. J Surg Res.

[CR76] Li S, Lv J, Li J, Zhao Z, Guo H, Zhang Y, Cheng S, Sun J, Pan H, Fan S, Li Z (2018). Intestinal microbiota impact sepsis associated encephalopathy via the vagus nerve. Neurosci Lett.

[CR77] Li F, Zhang B, Duan S, Qing W, Tan L, Chen S, Wang Y, Li D, Yang J, Tong J, Fang J, Le Y (2020). Small dose of L-dopa/Benserazide hydrochloride improved sepsis-induced neuroinflammation and long-term cognitive dysfunction in sepsis mice. Brain Res.

[CR78] Li C, Yu TY, Zhang Y, Wei LP, Dong SA, Shi J, Du SH, Yu JB (2020). Electroacupuncture improves cognition in rats with sepsis-associated encephalopathy. J Surg Res.

[CR79] Li S, Guo H, Xu X, Hua R, Zhao Q, Li J, Lv J, Li J (2021). Therapeutic methods for gut microbiota modification in lipopolysaccharide-associated encephalopathy. Shock.

[CR80] Liu J, Jin Y, Ye Y, Tang Y, Dai S, Li M, Zhao G, Hong G, Lu ZQ (2021). The neuroprotective effect of short chain fatty acids against sepsis-associated encephalopathy in mice. Front Immunol.

[CR81] Luo RY, Luo C, Zhong F, Shen WY, Li H, Hu ZL, Dai RP (2020). ProBDNF promotes sepsis-associated encephalopathy in mice by dampening the immune activity of meningeal CD4^+^ T cells. J Neuroinflammation.

[CR82] Mai SHC, Sharma N, Kwong AC, Dwivedi DJ, Khan M, Grin PM, Fox-Robichaud AE, Liaw PC (2018). Body temperature and mouse scoring systems as surrogate markers of death in cecal ligation and puncture sepsis. Intensive Care Med Exp.

[CR83] Maitland K, Kiguli S, Opoka RO, Engoru C, Olupot-Olupot P, Akech SO, Nyeko R, Mtove G, Reyburn H, Lang T, Brent B, Evans JA, Tibenderana JK, Crawley J, Russell EC, Levin M, Babiker AG, Gibb DM (2011). Mortality after fluid bolus in African children with severe infection. N Engl J Med..

[CR84] Maitland K, George EC, Evans JA, Kiguli S, Olupot-Olupot P, Akech SO, Opoka RO, Engoru C, Nyeko R, Mtove G, Reyburn H, Brent B, Nteziyaremye J, Mpoya A, Prevatt N, Dambisya CM, Semakula D, Ddungu A, Okuuny V, Wokulira R, Timbwa M, Otii B, Levin M, Crawley J, Babiker AG, Gibb DM, FEAST trial group (2013). Exploring mechanisms of excess mortality with early fluid resuscitation: insights from the FEAST trial. BMC Med.

[CR85] Manabe T, Heneka MT (2021). Cerebral dysfunctions caused by sepsis during ageing. Nat Rev Immunol.

[CR86] Matics TJ, Sanchez-Pinto LN (2017). Adaptation and validation of a pediatric sequential organ failure assessment score and evaluation of the sepsis-3 definitions in critically Ill Children. JAMA Pediatr..

[CR87] Mazeraud A, Righy C, Bouchereau E, Benghanem S, Bozza FA, Sharshar T (2020). Septic-associated encephalopathy: a comprehensive review. Neurotherapeutics.

[CR88] Michels M, Steckert AV, Quevedo J, Barichello T, Dal-Pizzol F (2015). Mechanisms of long-term cognitive dysfunction of sepsis: from blood-borne leukocytes to glial cells. Intensive Care Med Exp.

[CR89] Michels M, Ávila P, Pescador B, Vieira A, Abatti M, Cucker L, Borges H, Goulart AI, Junior CC, Barichello T, Quevedo J, Dal-Pizzol F (2019). Microglial cells depletion increases inflammation and modifies microglial phenotypes in an animal model of severe sepsis. Mol Neurobiol.

[CR90] Mo Y, Wang L, Ren M, Xie W, Ye X, Zhou B, Zhang A, Dai Q, Wang J (2021). Electroacupuncture prevents LPS- induced neuroinflammation via upregulation of PICK-TLR4 complexes in the microglia of hippocampus. Brain Res Bull.

[CR91] Mohamed El-Mashad G, Said El-Mekkawy M, Helmy ZM (2020). La escala pediátrica de evaluación del fallo multiorgánico secuencial (pSOFA): una nueva escala de predicción de la mortalidad en la unidad de cuidados intensivos pediátricos [Paediatric sequential organ failure assessment (pSOFA) score: A new mortality prediction score in the paediatric intensive care unit]. An Pediatr (Engl Ed)..

[CR92] Molnár L, Fülesdi B, Németh N, Molnár C (2018). Sepsis-associated encephalopathy: a review of literature. Neurol India.

[CR93] Moraes CA, Zaverucha-do-Valle C, Fleurance R, Sharshar T, Bozza FA, d'Avila JC (2021). Neuroinflammation in sepsis: molecular pathways of microglia activation. Pharmaceuticals (basel).

[CR94] Nwafor DC, Brichacek AL, Mohammad AS, Griffith J, Lucke-Wold BP, Benkovic SA, Geldenhuys WJ, Lockman PR, Brown CM (2019). Targeting the blood–brain barrier to prevent sepsis-associated cognitive impairment. J Cent Nerv Syst Dis.

[CR95] Orhun G, Esen F, Özcan PE, Sencer S, Bilgiç B, Ulusoy C, Noyan H, Küçükerden M, Ali A, Barburoğlu M, Tüzün E (2019). Neuroimaging findings in sepsis-induced brain dysfunction: association with clinical and laboratory findings. Neurocrit Care.

[CR96] Osca-Verdegal R, Beltrán-García J, Pallardó FV, García-Giménez JL (2021). Role of microRNAs as biomarkers in sepsis-associated encephalopathy. Mol Neurobiol..

[CR97] Pantzaris ND, Platanaki C, Tsiotsios K, Koniari I, Velissaris D (2021). The use of electroencephalography in patients with sepsis: a review of the literature. J Transl Int Med.

[CR98] Peng X, Luo Z, He S, Zhang L, Li Y (2021). Blood–brain barrier disruption by lipopolysaccharide and sepsis-associated encephalopathy. Front Cell Infect Microbiol.

[CR99] Pfister D, Siegemund M, Dell-Kuster S, Smielewski P, Rüegg S, Strebel SP, Marsch SC, Pargger H, Steiner LA (2008). Cerebral perfusion in sepsis-associated delirium. Crit Care.

[CR100] Pierrakos C, Attou R, Decorte L, Kolyviras A, Malinverni S, Gottignies P, Devriendt J, De Bels D (2014). Transcranial Doppler to assess sepsis-associated encephalopathy in critically ill patients. BMC Anesthesiol.

[CR101] Poggi C, Dani C (2018). Sepsis and oxidative stress in the newborn: from pathogenesis to novel therapeutic targets. Oxid Med Cell Longev.

[CR102] Poles MZ, Nászai A, Gulácsi L, Czakó BL, Gál KG, Glenz RJ, Dookhun D, Rutai A, Tallósy SP, Szabó A, Lőrinczi B, Szatmári I, Fülöp F, Vécsei L, Boros M, Juhász L, Kaszaki J (2021). Kynurenic acid and its synthetic derivatives protect against sepsis-associated neutrophil activation and brain mitochondrial dysfunction in rats. Front Immunol.

[CR103] Poli-de-Figueiredo LF, Garrido AG, Nakagawa N, Sannomiya P (2008). Experimental models of sepsis and their clinical relevance. Shock.

[CR104] Postelnicu R, Evans L (2017). Monitoring of the physical exam in sepsis. Curr Opin Crit Care.

[CR105] Punchak M, Hall K, Seni A, Buck WC, DeUgarte DA, Hartford E, Kelly RB, Muando VI (2018). Epidemiology of disease and mortality from a PICU in mozambique. Pediatr Crit Care Med.

[CR106] Reis PA, Alexandre PCB, D'Avila JC, Siqueira LD, Antunes B, Estato V, Tibiriça EV, Verdonk F, Sharshar T, Chrétien F, Castro-Faria-Neto HC, Bozza FA (2017). Statins prevent cognitive impairment after sepsis by reverting neuroinflammation, and microcirculatory/endothelial dysfunction. Brain Behav Immun.

[CR107] Ren C, Yao RQ, Zhang H, Feng YW, Yao YM (2020). Sepsis-associated encephalopathy: a vicious cycle of immunosuppression. J Neuroinflammation.

[CR108] Rittirsch D, Hoesel LM, Ward PA (2007). The disconnect between animal models of sepsis and human sepsis. J Leukoc Biol.

[CR109] Rivera-Lara L (2019). The role of impaired brain perfusion in septic encephalopathy. Crit Care.

[CR110] Rosenblatt K, Walker KA, Goodson C, Olson E, Maher D, Brown CH, Nyquist P (2020). Cerebral autoregulation-guided optimal blood pressure in sepsis-associated encephalopathy: a case series. J Intensive Care Med.

[CR111] Saito M, Fujinami Y, Ono Y, Ohyama S, Fujioka K, Yamashita K, Inoue S, Kotani J (2021). Infiltrated regulatory T cells and Th2 cells in the brain contribute to attenuation of sepsis-associated encephalopathy and alleviation of mental impairments in mice with polymicrobial sepsis. Brain Behav Immun.

[CR112] Samuels DC, Hulgan T, Fessel JP, Billings FT, Thompson JL, Chandrasekhar R, Girard TD (2019). Mitochondrial DNA haplogroups and delirium during sepsis. Crit Care Med.

[CR113] Sandquist MK, Clee MS, Patel SK, Howard KA, Yunger T, Nagaraj UD, Jones BV, Fei L, Vadivelu S, Wong HR (2017). High frequency of neuroimaging abnormalities among pediatric patients with sepsis who undergo neuroimaging. Pediatr Crit Care Med.

[CR114] Sanz D, D'Arco F, Robles CA, Brierley J (2018). Incidence and pattern of brain lesions in paediatric septic shock patients. Br J Radiol..

[CR115] Savi FF, de Oliveira A, de Medeiros GF, Bozza FA, Michels M, Sharshar T, Dal-Pizzol F, Ritter C (2021). What animal models can tell us about long-term cognitive dysfunction following sepsis: a systematic review. Neurosci Biobehav Rev.

[CR116] Shrum B, Anantha RV, Xu SX, Donnelly M, Haeryfar SM, McCormick JK, Mele T (2014). A robust scoring system to evaluate sepsis severity in an animal model. BMC Res Notes.

[CR117] Shulyatnikova T, Verkhratsky A (2020). Astroglia in sepsis associated encephalopathy. Neurochem Res..

[CR118] Sonneville R, de Montmollin E, Poujade J, Garrouste-Orgeas M, Souweine B, Darmon M, Mariotte E, Argaud L, Barbier F, Goldgran-Toledano D, Marcotte G, Dumenil AS, Jamali S, Lacave G, Ruckly S, Mourvillier B, Timsit JF (2017). Potentially modifiable factors contributing to sepsis-associated encephalopathy. Intensive Care Med.

[CR119] Spapen H, Nguyen DN, Troubleyn J, Huyghens L, Schiettecatte J (2010). Drotrecogin alfa (activated) may attenuate severe sepsis-associated encephalopathy in clinical septic shock. Crit Care.

[CR120] Su CM, Cheng HH, Tsai TC, Hsiao SY, Tsai NW, Chang WN, Lin WC, Cheng BC, Su YJ, Chang YT, Chiang YF, Kung CT, Lu CH (2014). Elevated serum vascular cell adhesion molecule-1 is associated with septic encephalopathy in adult community-onset severe sepsis patients. Biomed Res Int.

[CR121] Sui DM, Xie Q, Yi WJ, Gupta S, Yu XY, Li JB, Wang J, Wang JF, Deng XM (2016). Resveratrol protects against sepsis-associated encephalopathy and inhibits the NLRP3/IL-1β axis in microglia. Mediators Inflamm..

[CR122] Szatmári S, Végh T, Csomós A, Hallay J, Takács I, Molnár C, Fülesdi B (2010). Impaired cerebrovascular reactivity in sepsis-associated encephalopathy studied by acetazolamide test. Crit Care.

[CR123] Szöllősi D, Hegedűs N, Veres DS, Futó I, Horváth I, Kovács N, Martinecz B, Dénes Á, Seifert D, Bergmann R, Lebeda O, Varga Z, Kaleta Z, Szigeti K, Máthé D (2018). Evaluation of brain nuclear medicine imaging tracers in a murine model of sepsis-associated encephalopathy. Mol Imaging Biol.

[CR124] Takemoto R, Motomura Y, Kaku N, Ichimiya Y, Muraoka M, Kanno S, Tanaka T, Sakai Y, Maehara Y, Ohga S (2019). Late-onset sepsis and encephalopathy after bicycle-spoke injury: a case report. BMC Infect Dis.

[CR125] Tang G, Yang H, Chen J, Shi M, Ge L, Ge X, Zhu G (2017). Metformin ameliorates sepsis-induced brain injury by inhibiting apoptosis, oxidative stress and neuroinflammation via the PI3K/Akt signaling pathway. Oncotarget.

[CR126] Tauber SC, Djukic M, Gossner J, Eiffert H, Brück W, Nau R (2021). Sepsis-associated encephalopathy and septic encephalitis: an update. Expert Rev Anti Infect Ther.

[CR127] Tian M, Qingzhen L, Zhiyang Y, Chunlong C, Jiao D, Zhang L, Li W (2019). Attractylone attenuates sepsis-associated encephalopathy and cognitive dysfunction by inhibiting microglial activation and neuroinflammation. J Cell Biochem..

[CR128] Towner RA, Saunders D, Smith N, Towler W, Cruz M, Do S, Maher JE, Whitaker K, Lerner M, Morton KA (2018). Assessing long-term neuroinflammatory responses to encephalopathy using MRI approaches in a rat endotoxemia model. Geroscience..

[CR129] Tsuruta R, Oda Y (2016). A clinical perspective of sepsis-associated delirium. J Intensive Care.

[CR130] Vasiljevic B, Maglajlic-Djukic S, Gojnic M, Stankovic S, Ignjatovic S, Lutovac D (2011). New insights into the pathogenesis of perinatal hypoxic-ischemic brain injury. Pediatr Int.

[CR131] Visitchanakun P, Tangtanatakul P, Trithiphen O, Soonthornchai W, Wongphoom J, Tachaboon S, Srisawat N, Leelahavanichkul A (2020). Plasma miR-370-3P as a biomarker of sepsis-associated encephalopathy, the transcriptomic profiling analysis of microrna-arrays from mouse brains. Shock.

[CR132] Wang L, Liang Q, Lin A, Wu Y, Min H, Song S, Wang Y, Wang H, Yi L, Gao Q (2019). Borneol alleviates brain injury in sepsis mice by blocking neuronal effect of endotoxin. Life Sci.

[CR133] Wang P, Wang W, Hu Y, Li Y (2020). Prolonged soluble epoxide hydrolase reactivity in brain endothelial cells is associated with long cognitive deficits in sepsis. Mol Neurobiol.

[CR134] Wang J, Yang M, Xu H, Huang C, Xia Z, Cheng Y, Shu X, Li Y, Shi B, Qin C, Xiao S, Liu M, Tang W (2022). Diagnostic value of ONSD in sepsis associated encephalopathy of New Zealand rabbits. Brain Res Bull.

[CR135] Weiss SL, Peters MJ, Alhazzani W, Agus MSD, Flori HR, Inwald DP, Nadel S, Schlapbach LJ, Tasker RC, Argent AC, Brierley J, Carcillo J, Carrol ED, Carroll CL, Cheifetz IM, Choong K, Cies JJ, Cruz AT, De Luca D, Deep A, Faust SN, De Oliveira CF, Hall MW, Ishimine P, Javouhey E, Joosten KFM, Joshi P, Karam O, Kneyber MCJ, Lemson J, MacLaren G, Mehta NM, Møller MH, Newth CJL, Nguyen TC, Nishisaki A, Nunnally ME, Parker MM, Paul RM, Randolph AG, Ranjit S, Romer LH, Scott HF, Tume LN, Verger JT, Williams EA, Wolf J, Wong HR, Zimmerman JJ, Kissoon N, Tissieres P (2020). Surviving sepsis campaign international guidelines for the management of septic shock and sepsis-associated organ dysfunction in children. Pediatr Crit Care Med.

[CR136] Wijdicks EF, Bamlet WR, Maramattom BV, Manno EM, McClelland RL (2005). Validation of a new coma scale: the FOUR score. Ann Neurol.

[CR137] Wolff S, Klatt S, Wolff JC, Wilhelm J, Fink L, Kaps M, Rosengarten B (2009). Endotoxin-induced gene expression differences in the brain and effects of iNOS inhibition and norepinephrine. Intensive Care Med.

[CR138] Wu L, Feng Q, Ai ML, Deng SY, Liu ZY, Huang L, Ai YH, Zhang L (2020). The dynamic change of serum S100B levels from day 1 to day 3 is more associated with sepsis-associated encephalopathy. Sci Rep.

[CR139] Xie K, Zhang Y, Wang Y, Meng X, Wang Y, Yu Y, Chen H (2020). Hydrogen attenuates sepsis-associated encephalopathy by NRF2 mediated NLRP3 pathway inactivation. Inflamm Res.

[CR140] Xu XE, Li MZ, Yao ES, Gong S, Xie J, Gao W, Xie ZX, Li ZF, Bai XJ, Liu L, Liu XH (2020). Morin exerts protective effects on encephalopathy and sepsis-associated cognitive functions in a murine sepsis model. Brain Res Bull.

[CR141] Yan S, Gao M, Chen H, Jin X, Yang M (2019). Expression level of glial fibrillary acidic protein and its clinical significance in patients with sepsis-associated encephalopathy. Zhong Nan Da Xue Xue Bao Yi Xue Ban.

[CR142] Yang Z, Sun T (2020). Response to "optic nerve sheath diameter guided detection of sepsis associated encephalopathy". Crit Care.

[CR143] Yang Y, Liang S, Geng J, Wang Q, Wang P, Cao Y, Li R, Gao G, Li L (2020). Development of a nomogram to predict 30-day mortality of patients with sepsis-associated encephalopathy: a retrospective cohort study. J Intensive Care.

[CR144] Yin L, Chen X, Ji H, Gao S (2019). Dexmedetomidine protects against sepsis-associated encephalopathy through Hsp90/AKT signaling. Mol Med Rep.

[CR145] Yuan M, Yan DY, Xu FS, Zhao YD, Zhou Y, Pan LF (2020). Effects of sepsis on hippocampal volume and memory function. World J Emerg Med.

[CR146] Zayek M, Bhat J, Bonner K, Blake M, Peevy K, Jha OP, Gulati R, Bhat R (2020). Implementation of a modified neonatal early-onset sepsis calculator in well-baby nursery: a quality improvement study. Pediatr Qual Saf..

[CR147] Zenaide PV, Gusmao-Flores D (2013). Biomarkers in septic encephalopathy: a systematic review of clinical studies. Rev Bras Ter Intensiva.

[CR148] Zhang LN, Wang XT, Ai YH, Guo QL, Huang L, Liu ZY, Yao B (2012). Epidemiological features and risk factors of sepsis-associated encephalopathy in intensive care unit patients: 2008–2011. Chin Med J (engl).

[CR149] Zhang QH, Sheng ZY, Yao YM (2014). Septic encephalopathy: when cytokines interact with acetylcholine in the brain. Mil Med Res.

[CR150] Zhang LN, Ai YH, Gong H, Guo QL, Huang L, Liu ZY, Yao B (2014). Expression and role of neuroglobin in rats with sepsis-associated encephalopathy. Crit Care Med.

[CR151] Zhang W, Zhu L, An C (2020). The blood brain barrier in cerebral ischemic injury—disruption and repair. Brain Hemorrhages..

[CR152] Zhang L, Zhang X, Wu T, Pan X, Wang Z (2021). Isoflurane reduces septic neuron injury by HO-1-mediated abatement of inflammation and apoptosis. Mol Med Rep.

[CR153] Zhao YZ, Gao ZY, Ma LQ, Zhuang YY, Guan FL (2017). Research on biogenesis of mitochondria in astrocytes in sepsis-associated encephalopathy models. Eur Rev Med Pharmacol Sci.

[CR154] Zhao T, Xia Y, Wang D, Pang L (2019). Association between elevated serum tau protein level and sepsis-associated encephalopathy in patients with severe sepsis. Can J Infect Dis Med Microbiol.

[CR155] Zhao L, Li Y, Wang Y, Gao Q, Ge Z, Sun X, Li Y (2021). Development and validation of a nomogram for the prediction of hospital mortality of patients with encephalopathy caused by microbial infection: a retrospective cohort study. Front Microbiol.

[CR156] Zhong X, Xie L, Yang X, Liang F, Yang Y, Tong J, Zhong Y, Zhao K, Tang Y, Yuan C (2020). Ethyl pyruvate protects against sepsis-associated encephalopathy through inhibiting the NLRP3 inflammasome. Mol Med.

[CR157] Zhou R, Sun X, Li Y, Huang Q, Qu Y, Mu D, Li X (2019). Low-dose dexamethasone increases autophagy in cerebral cortical neurons of juvenile rats with sepsis associated encephalopathy. Neuroscience.

[CR158] Zhu SZ, Huang WP, Huang LQ, Han YL, Han QP, Zhu GF, Wen MY, Deng YY, Zeng HK (2016). Huperzine A protects sepsis associated encephalopathy by promoting the deficient cholinergic nervous function. Neurosci Lett.

[CR159] Zhu J, Zhang M, Han T, Wu H, Xiao Z, Lin S, Wang C, Xu F (2019). Exploring the biomarkers of sepsis-associated encephalopathy (SAE): metabolomics evidence from gas chromatography–mass spectrometry. Biomed Res Int.

[CR160] Zhu Y, Wang K, Ma Z, Liu D, Yang Y, Sun M, Wen A, Hao Y, Ma S, Ren F, Xin Z, Li Y, Di S, Liu J (2019). SIRT1 activation by butein attenuates sepsis-induced brain injury in mice subjected to cecal ligation and puncture via alleviating inflammatory and oxidative stress. Toxicol Appl Pharmacol.

[CR161] Ziaja M (2013). Septic encephalopathy. Curr Neurol Neurosci Rep.

[CR162] Zujalovic B, Mayer B, Hafner S, Balling F, Barth E (2020). AChE-activity in critically ill patients with suspected septic encephalopathy: a prospective, single-centre study. BMC Anesthesiol.

